# Non-coding RNAs: The link between maternal malnutrition and offspring metabolism

**DOI:** 10.3389/fnut.2022.1022784

**Published:** 2022-11-10

**Authors:** Yuan Zeng, Yifan Wu, Qian Zhang, Xinhua Xiao

**Affiliations:** Key Laboratory of Endocrinology, Ministry of Health, Department of Endocrinology, Peking Union Medical College Hospital, Peking Union Medical College, Chinese Academy of Medical Sciences, Beijing, China

**Keywords:** DOHaD, non-coding RNA, gestational diabetes mellitus, metabolic syndrome, obesity

## Abstract

Early life nutrition is associated with the development and metabolism in later life, which is known as the Developmental Origin of Health and Diseases (DOHaD). Epigenetics have been proposed as an important explanation for this link between early life malnutrition and long-term diseases. Non-coding RNAs (ncRNAs) may play a role in this epigenetic programming. The expression of ncRNAs (such as long non-coding RNA H19, microRNA-122, and circular RNA-SETD2) was significantly altered in specific tissues of offspring exposed to maternal malnutrition. Changes in these downstream targets of ncRNAs lead to abnormal development and metabolism. This review aims to summarize the existing knowledge on ncRNAs linking the maternal nutrition condition and offspring metabolic diseases, such as obesity, type 2 diabetes (T2D) and non-alcoholic fatty liver disease (NAFLD).

## Introduction

Metabolic syndrome (MetS) is defined as the clustering of obesity/overweight, glucose intolerance/insulin resistance (IR), dyslipidemia, and hypertension. Because MetS is a clinical manifestation, there is no common consensus on its criteria. It is highly associated with an increased risk of obesity, diabetes mellitus (DM), non-alcoholic fatty liver disease (NAFLD) and cardiovascular disease (1). The prevalence of MetS is high worldwide, with 34.7% in the United States (2) and 41% in Mexico (3). These numbers are expected to increase with the aging population and developing society. Obesity increases the incidence of cardiovascular disease (CVD) events (4), and CVD events are the leading cause of death and disability in patients with DM (5) and NAFLD (6). These complications have brought huge disease burden and economic burden to society (7). Obesity (8), type 2 diabetes (T2D) (9), and NAFLD (10, 11) interact with each other and are closely related to diet (12), inflammatory signals, intestinal bacteria, etc. Genetics together with environmental and lifestyle risk factors [such as sedentary lifestyle (13, 14), diet (15), and the intrauterine environment (16, 17)] are thought to be the main drivers in the pathogenesis of these metabolic diseases.

The association between malnutrition and increased risk of chronic diseases later in life is broadly known as the “Developmental Origins of Health and Diseases” (DOHaD). It can be dated back to 1986, when Prof. David Barker and his colleagues compared the differences in infant mortality rates (reflecting on nutrition early in life) and death in adults from ischemic heart disease and other leading causes in England and Wales, exploring the association between poor living standards and high mortality rates (18). Then, the famous Dutch Hunger Winter study found a higher risk of glucose intolerance (19) and obesity (20) among adults whose mothers were exposed to famine (1944–1945) during gestation compared to those never exposed to famine. Likewise, the Chinese famine study of 1959–1961 suggested that the famine experience in early life could substantially determine the risk of chronic diseases in later life (21), such as T2D (22). For example, adults around the age of 56 who had been exposed to the Chinese famine *in utero* were 1.5 times more likely to develop T2D than those who had not been exposed (23). Early life malnutrition can manifest as dietary restriction/overnutrition or obesity in mother, intrauterine growth retardation (IUGR) or low/high birth weight in fetus. Both undernutrition and overnutrition in mothers during pregnancy could exert profound and long-term effects [such as obesity (24), T2D (25), or NAFLD (26)] on the adult health of their offspring (27). For instance, IUGR animals (caused by uterine artery ligation) had changes in organ growth and development of islet β cells and insulin-sensitive tissues, and increased susceptibility to metabolic diseases such as DM later in life (28), which can be triggered by epigenetic mechanisms. Moreover, it has been shown that maternal malnutrition modulates miRNAs expression in offspring.

Epigenetics refers to a stable and heritable change in gene expression without any changes in DNA sequence. Therefore, epigenetic modifications such as DNA methylation, histone modifications or non-coding RNA have the potential to transmit environmental information from ancestors to the next generation and even across generations (29, 30). In this review, we mainly focus on the more discussed microRNAs (miRNAs) in non-coding RNA (ncRNAs). ncRNAs such as miRNAs, long non-coding RNAs (lncRNAs), and circular RNAs (circRNAs), have been previously considered evolutionary junk with no coding potential (31), but now it is identified as critical regulatory molecules that mediate cellular processes and functions (32, 33) and have coding potential (34). Moreover, ncRNAs regulate a series of biological processes (35), such as cell proliferation, differentiation, development and apoptosis; thus, their abnormal expression is associated with many human diseases, such as DM (36) and CVD (33). miRNAs (approximately 22 nucleotides) (37), lncRNAs (>200 nucleotides) (35) and circRNAs (single-stranded continuous loop structures) (38) have different lengths and modes of action. miRNAs negatively regulate target mRNAs by specifically binding to the 3’-untranslated region of target mRNAs (37). lncRNAs regulate gene expression mainly by *cis* or *trans* regulation [reviewed in Kopp et al. (39)]. However, circRNAs act as sponges, decoys, scaffolds mechanisms to regulate its target genes [reviewed in (40)].

miRNAs play a crucial role in growth (41) and metabolism during prenatal and postnatal period (42). miRNAs may serve to communicate between the fetuses and mothers (43). miRNAs in maternal plasma during pregnancy could predict fetal diseases, such as a small-for-gestational-age (SGA) infant (44, 45). At birth, measuring miRNA expression in placental or umbilical cord blood also predicts some risks of offspring metabolic disorders. Alterations of placental miRNA expression (epigenetic alterations) may serve as a record of *in utero* exposures (46), and certain miRNA species are largely unique to the placenta (47, 48). Therefore, miRNA expression profiles associated with SGA or macrosomia may be powerful predictors of metabolic disease risk later in life (49). In addition, the interaction of offspring diet after birth and maternal nutrition during pregnancy can lead to metabolic disorders in offspring later life, accompanied by changes in the expression of some miRNAs. This suggests that the programmed planning in fetuses exposed to adverse intrauterine environments is closely linked to ncRNA.

Therefore, the specific purpose of this review is to review the expression and function of known ncRNAs that may play a role in maternal malnutrition and offspring metabolic diseases (Tables 1–3) and thus to investigate the utility of these changes as biomarkers in aiding diagnosis and prognosis and in predicting treatment response.

**TABLE 1:** The role and changes of ncRNA in animal adverse intrauterine environment and late metabolic disorders.

Animal model	Maternal treatment	Sex and time	State of offspring	Source (offspring)	ncRNA	Putative target
Swiss mice (26)	HFD (4 weeks before mating ∼ birth)	Male and female; At birth	AMPKα2↓; Impaired hepatic lipid metabolism with aging	Liver	let-7↑	Prkaa2 (encoding AMPK α2)↓
Swiss mice (66)	HFD (pregnancy and lactation)	Male and female; 4 weeks old	Body weight and adipose tissue mass↑; Plasma cholesterol↑; Liver TG deposition↑; Lipogenesis↑; Glucose intolerant and insulin resistance↑	Liver	miR-370↑ controls miR-122↓	SCD1↑; AGPAT1↑; HMGCR↓; JNK ↑; CPT1a↓; ACADVL ↓
C57BL/6J mice (113)	LP diet during pregnancy and lactation	Male and female; 8 weeks old	Body weight and length at birth↓; β-cell mass↓; Impaired glucose tolerance and insulin secretion	Pancreas	miR-15b↑	Cyclin D1 and cyclin D2↓
C57BL/6J mice (108)	LP diet throughout pregnancy	Male and female; 3 months old	Normal newborn body weight, body length; blood glucose; Abnormal neonatal β cell fraction; Insulin levels at birth↓; Glucose intolerance and insulin sensitivity↑ at 6 weeks old	Pancreas	miR-199a-3p and miR-342↑	mTOR protein levels↓
Albino Wistar rats (78)	Streptozotocin administration at 2 days of age (F0)	Male; At birth	Hepatic steatosis	Liver and plasma	miR-122↓	PPARγ↑
		Female; At birth	Glycemia and insulinemia; Normal blood glucose at 3 months old	NA	NA	NA
Albino Wistar rats (78)	GDM(F1)	Male; At birth	Insulinemia; MMP-2 and CTGF levels↑; NO and LPS production↑; Glycemia and body weight at birth and also 5 months old↑	Plasma	miR-122↓	NA
		Female; At birth	Glycemia and insulinemia; birth weight↑; MMP-2 and CTGF levels↑	NA	NA	NA
Albino Wistar rats (79)	GDM(F1)	Male; At birth	TG↑; Cholesterol↑	Liver	miR-130↓	PPARγ↑
		Female; At birth	TG↓; Cholesterol↓; Free fatty acids↓; Phospholipids↓	Liver	miR-9↓	PPARγ↑
C57BL/6J mice (103)	Obesogenic diet (6 weeks before mating ∼ lactation)	Male; 8 weeks old	Serum insulin↑; Fasting insulin levels↑; Insulin resistance	eWAT	miR-126↑	IRS-1 protein↓; IRβ protein↓
C57BL/6J mice (104)	Obesogenic diet (6 weeks before mating ∼ lactation)	Male; 6 months old	eWAT amount↑; Altered adipocyte morphology; Risk of type 2 diabetes↑	eWAT	miR-126-3p↑	Lunapark↓; IRS-1 protein↓
SD rats (86)	50% global caloric restriction during the later half of pregnancy	Male and female; 3 weeks old	Weights↓; cell proliferation↑; adipogenesis↑; PPARγ, ADRP, and C/EBPα↑	BMSCs	miR-30d and miR-103↑	Wnt signaling↓
C57BL/6 mice (109)	LP12.5 diet	Male and female; 6–8 weeks old	Normal β-cell fraction and glucose tolerance; birth weight↓	NA	NA	NA
		Male; 12 weeks old	Insufficient pancreatic β-cell fraction; Glucose intolerance; Insulin sensitivity↓	Pancreas	miR-342↑; miR-143↑; miR-219↑	Genes involvd in insulin resistance and adipogenesis
C57BL/6 mice (109)	LP12.5 diet	Male; 12 weeks old	Weight gain↑; Glucose intolerance and insulin resistance	eWAT	miR 342↑; miR 143 NC	NA
C57BL/6 (109)	LP12.5 diet	Male and female; 48–51 weeks old	Mild hyperglycemia and glucose intolerance; Insulin resistance (female only); β-cell mass↑ and impaired insulin secretion (male only)	NA	NA	NA
SD rats (122)	LP diet throughout pregnancy	Male and female; 18 months old	Skeletal muscle insulin resistance	Skeletal muscle	miR-29a↑	PPARδ↓ and then PGC1α↓; GLUT4↓
SD rats (73)	Caloric restriction prenatally	Female; 3 weeks old	Birth weight↓; Plasma insulin and leptin levels↑; Dyslipidemic	Plasma	miR-122↑	Genes mediating lipid metabolism
SD rats (73)	Caloric restriction prenatally and postnatally	Female; 3 weeks old	Fatty acid oxidation↑; Fatty acid synthesis↓; Body, liver, skeletal muscle, pancreas and brown adipose tissue weights↓; Plasma IGF1, insulin, leptin, glucose, TG and HDL-C concentrations↓	Liver	miR-122↓	DGAT1↑; ALDO-A↑; BCKDK↑; FASN and HMGCR↓; CPT1α and PGC1α↑
SD rats (125)	Fructose water (gestation and lactation)	Male and female; 160 days old	Serum HDL-C levels↓; Insulin resistance	Liver	miR-29a↑ miR-130a↑	*Igf1*↓
SD rats (90)	20% fructose water (gestation and lactation)	Male; 160 days old	Serum HDL-C level↓	Liver	miR-206↑	Lxra↓
SD rats (87)	A 50% food-restricted diet (pregnancy)	Male; At birth and 10 weeks old	Body and liver weight↓ (at birth only); Body weight↓; FOXO1 and PPARγ↑; TG content↑ (at 10 weeks old only)	Liver	miR-370-3p↓ (at birth only) miR-181a-5p↓	Targets for miR-181a-5p: SIRT1↑; KLF6 ↑

ACADVL, acyl-CoA dehydrogenase very long chain; ADRP, adipocyte differentiation-related protein; AGPAT1, 1-acylglycerol-3-phosphate *O*-acyltransferase 1; ALDO-A, aldolase A; AMPK, AMP-activated protein kinase; BCKDK, branched chain ketoacid dehydrogenase kinase; BMSCs, bone marrow-derived mesenchymal stem cells; C/EBPα, CCAAT/enhancer-binding protein α; CPT1a, carnitine palmitoyltransferase 1a; CTGF, connective tissue growth factor; DGAT1, diacylglycerol *O*-acyltransferase 1; eWAT, epididymal white adipose tissue; FASN, fatty acid synthase; FOXO1, forkhead box protein O1; GLUT4, glucose transporter 4; HDL-C, high-density lipoprotein-cholesterol; HMGCR, 3-hydroxy-3-methylglutaryl-CoA reductase; IGF1, insulin-like growth factor-1; IRS-1, insulin receptor substrate 1; IRβ, insulin receptor-beta; JNK, c-Jun N-terminal kinase; KLF6, krüppel-like factor 6; LP 12.5, the low protein exposure model during the last 7 days of pregnancy; LP diet, low-protein diet; LP0.5, a low protein diet throughout pregnancy; LP12.5, a low protein diet during the last week of pregnancy; LPS, lipopolysaccharide; Lxra, liver X receptor alpha; MMP-2, matrix metalloproteinase-2; mTOR, mechanistic target of rapamycin; NO, nitric oxide; SD rats, Sprague-Dawley rats; SIRT1, Sirtuin-1; SREBP-1c, sterol regulatory element-binding protein 1C; TG, triglyceride. ↑, increase; ↓, decrease; NC, no change; NA, not available.

**TABLE 2:** ncRNA involved in influencing the birth weight of human offspring.

Region	Pregnancy status	Source (newborns)	ncRNA name	Putative target	Potential role
México (102)	Macrosomia vs. adequate birth weight	Dried blood spots	miR-29a-5p, miR-126-3p, miR-221-3p, and miR-486-5p↑	Participated in FOXO and PI3K/AKT signaling pathways; Involved in carbohydrate metabolism	Involved in cell cycle, proliferation, apoptosis and metabolism; Associated with obesity, diabetes, and cardiovascular diseases
Shenyang, Liaoning, China (163)	Macrosomia (*n* = 25)	Placenta (*n* = 50)	circRNA-SETD2↑	miR-519a/PTEN axis	Regulated HTR8/SVneo cell proliferation and invasion
Nanjing, Jiangsu, China (141)	GDM-induced macrosomia (*n* = 32)	Umbilical cord blood (*n* = 79)	lncRNA RP11-290L1.3↑	PPARγ, SREBP-1c, and FASN↑; Involved metabolic pathways, such as insulin signaling pathway and MAPK signaling pathway	Involved in fat accumulation induced by GDM
Shenyang, China (148)	IUGR (*n* = 30) and LGA (*n* = 30)	Placenta (*n* = 90)	miR-518b↓ miR-519a↑	Target for miR-519a: Gab1; PTEN; HIF-1α	Regulated placental development and trophoblast proliferation and invasion; Associated with birth weight
Rhode Island, US (49)	SGA	Placenta (*n* = 107)	miR-16↓; miR-21↓	Target for miR-16: BCL-2; Target for miR-21: PTEN	Associated with poor fetal growth
Jiangsu, China (151)	Macrosomia (*n* = 35)	Placenta (*n* = 70)	miR-21↑; miR-16 NC	Eight possible pathways by bioinformatics analysis, such as PI3K/AKT, P53, MAPK, HIF-1, TGF-β, Wnt, Jak-STAT, and mTOR signaling pathway	Increased the risk of macrosomia
Wenzhou, China (115)	Macrosomia (*n* = 67)	Placenta (*n* = 131)	miR-21↑; miR-143↓; miR-16 NC	MAPK signaling pathway	Influenced the risk of macrosomia

FASN, fatty acid synthase; FOXO, forkhead box protein O; Gab1, Grb2 –associated binder 1; GDM, gestational diabetes mellitus; HIF-1α, hypoxia-inducible transcription factors 1α; IUGR, intrauterine growth retardation; LGA, large for gestational age; MAPK, mitogen-activated protein kinase; mTOR, mechanistic target of rapamycin; PI3K/AKT, phosphatidylinositol 3-kinase/protein kinase B; PPARγ, peroxisome proliferator-activated receptor γ; PTEN, phosphatase and tensin homolog deleted on chromosome 10; SREBP-1c, sterol regulatory element-binding protein 1C. ↑, increase; ↓, decrease; NC, no change.

**TABLE 3:** Changes in ncRNA and its potential role in human IUGR pregnancy.

Region	Pregnancy status	ncRNA	Source	Putative target	Result/Potential roles
Chongqing, China (169)	IUGR pregnancy (*n* = 20)	lncRNA H19↓	Placenta (*n* = 40)	Regulate the PI3K/AKT-mTOR and MAPK/ERK-mTOR pathways; Bind to miR-18a –5p, which targets interferon regulatory factor-2	Promoted autophagy; Suppressed cell proliferation and invasion
Guangdong, China (174)	IUGR pregnancy (*n* = 37)	Circular RNA hsa_circ_0081343↓	Placenta (*n* = 64)	The miR-210-5p/DLX3 axis	Regulated the migration, invasion, and apoptosis of HTR-8 cells
Nagasaki, Japan (146)	IUGR pregnancy (*n* = 45)	miR-518b, miR-1323, miR-516b, miR-515-5p, miR-520h, miR-519d and miR-526b↓	Placenta (*n* = 95)	NA	Identified as IUGR placenta-specific microRNAs
Tianjin, China (175)	IUGR pregnancy	miR-212-3p↑	Placenta	Placental growth factor↓	Promoted cell proliferation and invasion
California, US (143)	IUGR pregnancy (*n* = 5)	miR-10b↑; miR-363↑; miR-149↑	Placenta (*n* = 37)	Target for miR-10b: *E*-cadherin↑; Krüppel-like factor 4↓ Target for miR-363: SNAT1 and SNAT2↓ Target for miR-149: L-type amino acid transporter 2↓	Related to the development of IUGR
	SGA pregnancy (*n* = 11)				
Nanjing, China (153)	IUGR pregnancies (*n* = 21)	miR-141↑	Placenta (*n* = 55)	Pleomorphic adenoma gene 1↓-IGF↓; E2F transcription factor 3 protein↓; MAPK signaling pathway; Wnt signaling pathway	Related to the development of IUGR
Guangzhou, China (144)	IUGR pregnancy (*n* = 19)	miR-424↑	Placenta (*n* = 39)	ERRγ and HSD17B1 (an human enzyme that catalyzes the formation of highly active estradiol)↓	Related to the development of IUGR

ERRγ, estrogen-related receptor γ; HSD17B1, 17 beta-hydroxysteroid dehydrogenase type 1; mTOR, mechanistic target of rapamycin; DLX3, Distal-less 3; IUGR, intrauterine growth retardation; MAPK, mitogen-activated protein kinase; PI3K/AKT, phosphatidylinositol 3-kinase/protein kinase B; SNAT1 and SNAT2, sodium-coupled amino acid transporter 1 and 2; SGA, small-for-gestational-age. ↑, increase; ↓, decrease.

## Role of miRNAs in lipid metabolism in offspring

### miR-122

As a mammalian liver-specific microRNA, microRNA-122 (miR-122) is expressed abundantly in the liver, accounting for approximately 70% of all cloned miRNAs in mouse (50) and human adult (51) livers. It is highly involved in liver physiology and diseases such as metabolic disorders (52, 53) by regulating genes such as *Klf6* (krüppel-like factor 6) [reviewed in (54)]. After the production in the liver, miR-122 can be transported into the blood, thus influencing distant organs. Generally, reduction or loss function of liver miR-122 can result in deleterious consequences such as NAFLD and the onset and progression of tumors, while circulating miR-122 levels were often increased as a sensitive biomarker for liver injury [reviewed in (55)]. Artificial expression of miR-122 protects mice and humans from liver disease induced by ethanol (56). However, there is some controversy. Some studies found that the inhibition of miR-122 in a high-fat diet (HFD) animal model protected the liver from NAFLD and suppressed lipogenesis (57), which is related to a complex regulatory network (58). High expression levels of circulating miR-122 increased the risk of obesity and might be a potential biomarker (59, 60), whereas low circulating miR-122 levels reflected diet-induced weight loss (61). Additionally, serum miR-122 showed the great diagnostic accuracy for NAFLD in Filipinos (62) and in the female West Virginia population (63), with an approximate area under the receiver operating characteristic (AUROC) of ≥0.85 and a significant *P*-value, which are comparable to serum ALT (AUROC = 0.832, *P* = 0.001) (62). Zhang et al. systematically reviewed the overall trend of decreased hepatic miR-122 expression and increased serum miR-122 expression in NAFLD patients (64).

**FIGURE 1:**
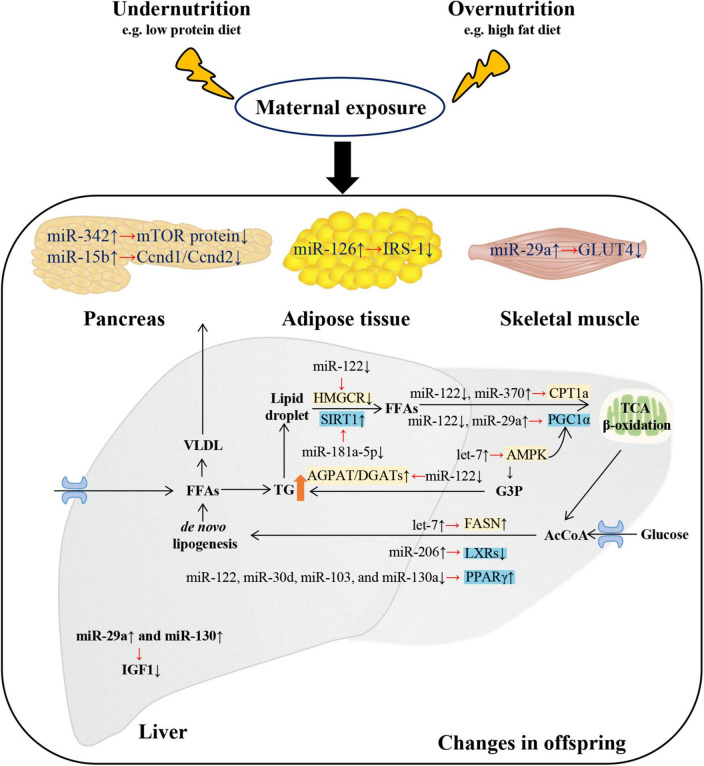
Changes and putative targets of microRNAs (miRNAs) on metabolism-related tissues of metabolic syndrome of offspring exposed to maternal malnutrition. Metabolic disturbances in offspring exposed to maternal malnutrition were regulated by miRNAs. Disorders of glucose metabolism mainly involved four tissues: liver, pancreas, adipose tissue, and skeletal muscle. In the liver of offspring exposed to maternal fructose intake, the increase of miR-29a and miR-130 resulted in the decrease of hepatic insulin-like growth factor-1 (Igf1) mRNA and protein expression. In pancreas of offspring exposed to maternal low protein diet, the decline of cyclin D1 (Ccnd1)/cyclin D2 (Ccnd2) and mTOR protein levels were due to the rise of miR-15b and miR-342 respectively. In white adipose tissue of mice offspring exposed to maternal HFD-induced obesity, the insulin receptor substrate 1 (IRS-1) was decreased by miR-126. In the skeletal muscle of IUGR rat, the increase of miR-29a resulted in the decrease of glucose transporter 4 (GLUT4). As for lipid metabolism, it mainly involved liver tissue. Free fatty acids (FFAs) produced from white adipose tissue and dietary sources can enter hepatocyte. And FFAs are usually esterified to triacylglycerol (TG) and then packaged as VLDL (very low-density lipoprotein) for export or stored as intracellular lipid droplets. MicroRNAs regulate the hydrolysis of TG back to FFAs and then into the process of mitochondrial β-oxidation (by regulating various coactivators or nuclear receptors such as sirtuin-1 (SIRT1), PPARγ co-activator 1α (PGC1α), and genes such as CPT1a (carnitine palmitoyltransferase 1a). In addition, miRNAs promoted *de novo* lipogenesis by regulating genes (such as Fasn) and transcription factors [such as liver X receptors (LXRs) and peroxisome proliferator-activated receptor γ (PPARγ)]. The overall metabolic trend is TG accumulation and hyperglycemia. G3P, glycerol-3-phosphate; AGPAT, acyl-CoA acylglycerol-3-phosphate acyltransferase; AcCoA, acetyl-CoA; DGAT, diacylglycerol acyltransferase. ↑/↓ showed the expression changes of miRNAs, genes and proteins.

Maternal nutrition regulates miRNA expression in offspring and then affect their metabolism (65). Benatti et al. (66) measured microRNA expression at 4 weeks old in the liver of the offspring of mothers on a HFD or a standard diet during pregnancy and lactation. They found decreased expression of hepatic miR-122 at 4 weeks in pup mice exposed to maternal HFD, and some metabolic changes such as glucose intolerance and insulin resistance, increased plasma cholesterol, triglyceride (TG) and non-esterified fatty acid (NEFA). At the time of sample collection (4 weeks old), pro-inflammatory pathways activated, TG content deposited and vacuoles containing lipids existed in liver. In addition, 1-acylglycerol-3-phosphate *O*-acyltransferase 1 (AGPAT1) mRNA and stearoyl-CoA desaturase 1 (SCD1) mRNA involved in TG synthesis were increased through the regulation of miR-122 (Figure 1) (66). Indeed, AGPAT1 (67, 68) and SCD1 (69) were shown to be targets of miR-122, and their abnormal expression caused lipid metabolism disorders (70, 71). And one of the possible mechanisms of maternal HFD feeding leading to NAFLD in offspring is up-regulated SCD1 (72). Interestingly, while plasma cholesterol increased, 3-hydroxy-3-methylglutaryl-CoA reductase (*Hmgcr*) mRNA content was reduced in HFD offspring (HFD-O) (66). HMGCR is a rate-limiting enzyme that catalyzes *de novo* cholesterol synthesis *in vivo*, so the activity of HMGCR affects the level of cholesterol. Benatti and his colleagues suggested that increased plasma cholesterol was caused by breast milk being affected by HFD during lactation (66). However, in the offspring of other diet-induced rat models (such as caloric restriction), HMGCR also decreased with the reduction of miR-122 (73). A study investigating the mechanism that miR-122 modulates hepatitis C virus RNA expression in humans found that inhibition of miR-122 reduced HCV and *HMGCR* RNA independently with the effect on *HMGCR* transcription rates (74). These results indirectly indicate that the decrease in miR-122 expression caused by maternal HFD might regulate the increase in AGPAT1 and SCD1 mRNA and the decrease in HMGCR mRNA in HFD-O mice, leading to lipid metabolism disorders. This finding sheds a light on therapeutic targets, such as reducing SCD1 expression to prevent diet-induced obesity in animals (75).

The concept that maternal body composition and diet affect the health of future generations is gaining ground (76, 77). In a 2020 study (78), neonatal rats that developed mild diabetes after streptozotocin administration were considered as F0, and then these adult females were mated with control males to produce F1, who developed gestational diabetes mellitus (GDM), and then F2 (F1 females mated with control males). Fornes et al. found that the levels of miR-122 in the liver and plasma were reduced in parallel to an increase of peroxisome proliferator-activated receptor γ (PPARγ) in the livers from male fetuses of F0 and F1 at birth (Figure 1) (78, 79). At the same time, the liver is in a prooxidant/proinflammatory and lipid accumulating state. The increased PPARγ levels linking to the lipid accumulation was sex-dependent (79), because a reduction in miR-122 was not found in female fetuses (78, 79). Thus, miR-122 and the PPARγ pathway are important in metabolic diseases in offspring. A maternal diet enriched in olive oil (rich in PPAR ligands) is beneficial for preventing the rise of the proinflammatory (such as tumor necrosis factor-α) and profibrotic markers (matrix metalloproteinases) in placentas of GDM rats and also for the fetuses (80). The same study team offered maternal olive oil supplementation during the gestation of F1 (81) under the same experimental model described above. They found that the maternal diet enriched in PPAR ligands throughout gestation prevented the reduced expression of miR-122 (78) and the increased levels of triglycerides, cholesterol and PPARγ in the livers from male fetuses of GDM rats (81). In conclusion, miR-122 might regulate PPARγ participating in the metabolic process of male offspring of GDM mice.

Maternal nutrition did affect the expression of liver miRNAs (82). Dai et al. (73) designed three experimental models, i.e., prenatal exposure (IUGR), postnatal exposure (PNGR), or both (IPGR) exposed to caloric restriction in female rats, to explore the role of liver miR-122. They found decreased miR-122 expression and a significant loss of liver, pancreas and skeletal muscle weight in PNGR and IPGR at 3 weeks old. At the same time, increased PPARγ coactivator 1α (PGC1α)/carnitine palmitoyltransferase 1a (CPT1a, mediating fatty acid oxidation) expression and reduced fatty acid synthase (FASN)/HMGCR (mediating fatty acid synthesis) expression were found in the liver in PNGR and IPGR (Figure 1) (73). In addition, the reduction in miR-122 also influenced other fatty acid-metabolizing genes such as diacylglycerol *O*-acyltransferase 1 (DGAT1), aldolase A (ALDO-A) and branched chain ketoacid dehydrogenase kinase (BCKDK) (73). The normal hepatic miR-122 but high circulating miR-122 accompanied by high circulating TG, fatty acid and insulin concentrations in IUGR reflected the catch-up growth, so the organ weights of IUGR groups exposed to adequate postnatal nutrition were not different from the control at 3 weeks old (73).

### miR-30d

Runt related transcription factor 2 (RUNX2), a transcription factor strongly repressing adipogenesis, is one of the targets of miR-30d. Overexpression of miR-30d affected mesenchymal transition and osteogenic ability of human umbilical vein endothelial cells by reducing RUNX2 (83). Thus, in humans, miR-30d can stimulate adipogenesis via the reduction of RUNX2 (84). Additionally, miR-30d is elevated in abdominal adipose tissue from subjects with obesity and diabetes, which suggests its possible role in adiposity and IR (85). Gong et al. (86) found that key adipogenic miRNAs, such as miR-30d and miR-103, were significantly increased in bone marrow-derived mesenchymal stem cells (BMSCs) of the IUGR rats (caused by maternal food restriction) at 3 weeks of age, while pups had low body weight at that time. Notably, an overall upregulated PPARγ but downregulated Wnt (such as RUNX2) signaling profile was detected (86), which suggests strong adipogenesis. The conclusion that miR-30d may be involved in catch-up growth of offspring exposed to maternal nutrient restriction (MNR, 50% global caloric restriction) should be considered with caution, as only one literature has described this result.

### miR-181a-5p

Zhu et al. compared the differentially expressed miRNAs between the liver of MNR offspring and of the control group and then verified the miRNAs using RT-qPCR. Finally, they chose miR-181a-5p (which is the most markedly downregulated miRNAs and also related to metabolism) to testify that maternal nutrition regulate miRNAs in early life of offspring (87). They found that miR-181a-5p was downregulated in the liver of MNR offspring at the age of 1 day and persisted until 10 weeks, as was body weight. At 10 weeks of age, hepatic TG content increased while body weight remained low. And miR-181a-5p upregulated sirtuin-1 (SIRT1, an NAD-dependent deacetylase) and Klf6 (a transcription factor of the zinc finger family) (Figure 1) (87). Consistent with a previous study, increased SIRT1 expression was also found in the liver of MNR offspring, which may lead to an increase in total cholesterol (88). However, in offspring exposed to maternal HFD, SIRT1 was downregulated in the liver (89). Importantly, SIRT1 overexpression in HFD offspring could improve lipid metabolism and even glucose metabolism (89). This may be a potential therapeutic target.

### miR-206

Yamazaki et al. (90) found that exposure to maternal fructose in early life caused a reduction in liver X receptor alpha (Lxrα) with a connection to the increase in miR-206 in the liver of offspring (Figure 1). Previously, Lxrα was also identified as the target of miR-206 (91), and the delivery of miR-206 into the livers could also reduce the expression of Lxrα (92). Therefore, the interaction between miR-206 and Lxrα might contribute to the decreased serum HDL-C in offspring. Similarly, due to altered expression of Lxrα, IUGR fetuses (F1, caused by *in utero* under nutrition) that developed obesity and glucose intolerance with aging influenced the expression of lipogenic genes in the livers of F2 mice (93). However, miR-206 was decreased in the livers of mice fed an HFD, and increasing miR-206 in the liver played a protective role in inhibiting lipid production (92). In addition, the increase in Lxrα could protect against hepatic steatosis (94). This is contradictory, but it also shows the multidirectional regulation of miR-206.

### miR-370

miR-370 was upregulated in the liver of offspring exposed to maternal HFD (66) but downregulated in the liver of offspring exposed to MNR (87) (Table 1). miR-370 indirectly activates adipogenic genes through miR-122 (as we discussed before) and directly downregulates CPT1a (carnitine palmitoyltransferase 1a), which controls rate-limiting steps in fatty acid β oxidation (95). Recently, it has been reported that miR-370 is also increased in the livers of mice fed an HFD and promotes NAFLD development by regulating miR-122 and let-7 and their targets such as CPT1a and Prkaa2 (protein kinase AMP-activated catalytic subunit alpha 2) (96). These findings suggest the importance of miR-370 changes in offspring exposed to nutrition stress.

## Role of miRNAs in glucose metabolism in offspring

### miR-126

miR-126 is abundantly expressed in endothelial cells and has been identified to regulate angiogenesis and vascular integrity. Links between endothelial miR-126 and T2D have been widely discussed. miR-126 appears to fulfill a critical role in the prediction and diagnosis of T2D, as several clinical studies have found that its expression in plasma is reduced significantly in patients with T2D (97, 98), and lower miR-126 levels correlated with diabetic complications such as thromboembolic events (99) and cardiac microangiopathy (100). Moreover, miR-126 plays a protective role in vascular injury and hypoxia through the target FOXO (forkhead box protein O) and the phosphatidylinositol 3-kinase/protein kinase B (PI3K/AKT) pathway [reviewed in Pishavar et al. (101)]. In addition, reporting the upregulated expression of miR-126 in dried blood spots of macrosomia, the researchers further demonstrated with biological analysis that high levels of miR-126 were associated with a higher risk of obesity and diabetes associated with macrosomia, with a putative target of FOXO and the PI3K/AKT pathway (102).

Furthermore, the targets of miR-126 in adipose tissue are insulin receptor substrate 1 (IRS-1) (103) and Lunapark (104). The former is the main substrate of insulin receptor tyrosine kinase and insulin-like growth factor 1 (IGF1), and the latter is a conserved membrane protein that stabilizes new three-way connections in the endoplasmic reticulum (105), so their reduction may impair glucose tolerance. The expression of miR-126 was upregulated in the epididymal white adipose tissue (eWAT) of male rat offspring exposed to maternal obesity induced by diet at 8 weeks old (103) and even at 6 months old (104), and its augmented expression targeted the decreasing levels of IRS-1 protein. Fernandez-Twinn et al. (103) hypothesized that male offspring exposed to maternal diet-induced obesity may drive IR (eventually T2D) in later life, which was associated with the reduction in IRS-1 targeted by miR-126, because at 8 weeks old, male offspring did not exhibit an obese phenotype but exhibited decreased levels of IRS-1 protein. In 2021, the same research group, de Almeida-Faria et al. (104), found that in male offspring exposed to maternal obesity during pregnancy, miR-126-3p could directly decrease the expression of IRS-1 and Lunapark. Therefore, these results have suggested that maternal diet-induced obesity leads to IR in offspring by the upregulation of miR-126. de Almeida-Faria et al. (106) also found that feeding an obesogenic diet after weaning resulted in increased IRS-1 degradation, which suggests that exposure to obese individuals *in utero* combined with a postweaning obesogenic diet could significantly increase the risk of T2D. Moreover, the expression of miR-126 also changed in brown fat deposition and lipid metabolism of adult male offspring exposed to maternal high-sucrose diet (107). The reasons for this was unclear, but probably due to variation in the Zbtb16 gene (107).

### miR-342

Alejandro et al. found that in the pancreas of offspring of mothers fed a low protein (LP) diet throughout pregnancy (LP0.5) (108) or during the last 7 days of pregnancy (LP12.5) (109), miR-342 were upregulated. The offspring had glycometabolic disorders, such as glucose intolerance and IR, at 12 weeks of age and reduced insulin levels at birth (108, 109). The decreased mechanistic target of rapamycin (mTOR) protein levels regulated by miR-342 can explain the abnormal pancreas β cell mass and function in offspring (108), causing permanent changes that may contribute to the MetS later in life. Consistent with a previous study, miR-342 may be involved in pancreatic β-cell differentiation and maturation [reviewed in Kaviani et al. (110)]. Notably, miR-342 (–/–) mice fed with HFHS chow had lower blood glucose levels and fat weight and higher insulin and leptin sensitivity, which suggested that the loss of miR-342 can protect against obesity and diabetes (111). This suggested the therapeutic role of miR-342.

### miR-15b

miR-15b is known to induce the apoptosis of rat activated pancreatic stellate cells *in vitro*. Recently, there was a report that miR-15b-5p can target cyclin D1 and cyclin D2 to attenuate pancreatic β-cell proliferation and insulin secretion (112). Su et al. (113) found that miR-15b was increased in the islets of LP offspring at 8 weeks of age, impairing glucose metabolism by targeting cyclin D1 and cyclin D2 (Table 1). LP mouse offspring presented an inhibited pancreatic β-cell mass/proliferation and insulin secretion accompanied by low body weight. Additionally, the use of miR-15b inhibitors could rescue impaired glucose metabolism. This finding indicates the importance of miR-15b in glucose metabolism and even T2D development. A clinical study found a significant increase in circulating miR-15b in obese children and adults with T2D (114). Additionally, miR-15b levels could differentiate between T2D patients and healthy controls (AUROC = 0.969) (114). Again, this gives us a further understanding of the role of miR-15b in predicting future T2D risk.

### miR-143

miR-143 was increased in the pancreas of LP12.5 male offspring, suggesting a role in insulin secretion and resistance (109). Conversely, miR-143 was decreased in placenta of macrosomia, which may target the mitogen-activated protein kinase (MAPK) signaling pathway to be involved in subsequent metabolic disorders (115). miR-143 is involved in the development of human T2DM by inhibiting the insulin-AKT pathway (116). Therefore, miR-143 may be an underlying treatment target.

### miR-199a-3p

miR-199a-3p resulted in increased pancreas β cell apoptosis in T2D mice (117). And it was increased in the adult LP0.5 pancreas and could target the mTOR signaling pathway, thus impairing pancreatic β-cell and causing metabolic disturbance (108). A meta-analysis confirmed that miR-199a-3p was abnormally modulated in animal models of diabetes (118).

## Role of miRNAs in both glucose and lipid metabolism in offspring

### miR-29a

Generally, a protective role is played by miR-29a in glucolipid metabolism. *In vitro*, miR-29a inhibited MIN6 (the mouse insulinoma cell line) proliferation and insulin secretion (119) and mitigated high glucose-induced oxidative injury (120). Moreover, miR-29a can improve HFD-induced obesity and liver fibrosis (121). Zhou et al. (122) found that miR-29a was significantly upregulated in muscle samples from IUGR rats at 18 months of age. In their previous study, IUGR rats weighed more than controls and IR was observed in skeletal muscle at 18 months of age (123). Overexpression of miR-29a in C2C12 (skeletal muscle cell line) decreased the levels of glucose transporter 4 (GLUT4) and also downregulated its target gene peroxisome proliferator-activated receptor δ (PPARδ), thereby reducing PGC1α expression (Figure 1) (122). In contrast, overexpression of PGC1α can mediate fatty acid oxidation, attenuating HFD-induced hepatic steatosis (124). Interestingly, in clinical studies, Ortiz-Dosal et al. (102) quantified miRNAs associated with metabolic diseases in dried blood spots of newborns with adequate birth weight, low birth weight (LBW) or macrosomia. They determined that miR-29a-5p was also upregulated in macrosomia, which may explain the increased risk of obesity and diabetes associated with macrosomia (102). Consistent with a previous study, Munetsuna et al. found that maternal fructose intake resulted in an increase in miR-29a in the livers of the offspring at 160 days of age, which decreased *Igf1* mRNA expression. Moreover, reduced IGF1 expression may induce IR and impair hepatic function (125). However, no impaired IR or decreased high-density lipoprotein cholesterol (HDL-C) levels were observed at 60 days of age, even with the increase in miR-29a (125). Therefore, it is possible to use the expression of miRNAs to predict the metabolic state later in life. These findings suggest that miR-29a is broadly involved in glucolipid metabolism in offspring.

### miR-130a

miR-130a/b can inhibit *de novo* lipogenesis, but enhance lipolysis (126) and regulate insulin sensitivity (127), thus being involved in metabolic diseases such as liver steatosis and T2D. Munetsuna et al. (125) found that miR-130a was increased in the liver of offspring exposed to maternal fructose consumption at 60 days of age and persisted until 160 days of age, resulting in a decrease in its target *Igf1* and thus affecting glucolipid metabolism. Conversely, Forness et al. (79) found that miR-130 was decreased at Day 21 of gestation in the liver of male fetuses of GDM mothers while PPARγ levels were increased (Figure 1). Additionally, the body weight and TG were increased in the male fetuses of GDM mothers. It has been reported that a decrease in miR-130a can target genes such as PPARγ to promote lipid accumulation and even result in NAFLD (128). These results suggest that the reduction of miR-130 targeting PPARγ increases lipid accumulation in male fetal GDM rats and even causes NAFLD in adulthood. Another role of miR-130a in NAFLD is perpetuating fibrogenesis (129). Additionally, in human cirrhosis patients, the expression of miR-130a-3p was significantly decreased in monocyte-derived macrophages (130). Therefore, miR-130a might be used as a biomarker for NAFLD in the clinic.

### Let-7

As one of the first known miRNAs, let-7 is often presented as a tumor suppressor [reviewed in Lee et al. (131)]. We are more interested in its regulatory role in metabolic processes such as controlling glucose metabolism and insulin sensitivity and inducing autophagy under nutrient deficiency conditions [reviewed in Jiang et al. (132)]. Meanwhile, the knockdown of let-7 with an anti-miR might provide a therapy to treat metabolic diseases such as T2D (133). Recently, Simino et al. proposed that let-7a was upregulated in livers at the delivery day and decreased the levels of AMP-activated protein kinase α2 (AMPKα2) protein and Lin28a, causing the metabolic disturbances of offspring from obesity-prone HFD-fed dams (OP-O) (26). They previously reported that OP-O mice presented higher hepatic TG, serum glucose/insulin and cholesterol levels, diminished Prkaa2 (the gene that encodes AMPKα2) and upregulated *Fasn* and *Srebf1* (sterol regulatory element binding transcription factor 1) after weaning (134). This indicated that OP-O mice were early prone to developing metabolic disturbances, such as NAFLD. In addition, they showed that let-7 anti-miR transfection in hepatocytes can prevent the fat accumulation. The activation of the AMPK complex inhibits targets such as HMGCR, acetyl-CoA carboxylase (ACC), and glycogen synthase thus regulating fatty acid and sterol synthesis and glycogen storage [reviewed in Herzig et al. (135)]. Thus, the decrease in AMPKα2 targeted by let-7 might explain the early metabolic disorders in offspring and even the susceptibility to NAFLD in the future (Figure 1).

## Role of ncRNAs in abnormal birth weight in offspring

The birth weight of offspring and ncRNAs derived from placenta and umbilical cord blood have a significant relationship. Back in 2011, a study exploring the relationship between placental miRNAs expression profile and birth weight found that miRNAs are a good predictor of birth weight (49). Recently, cohort studies from Sweden, Belgium and USA showed that miRNAs (136–138) and lncRNAs (139) derived from placentas were associated with abnormal birth weight. miR-191-3p from umbilical cord blood reliably differentiated LBW (*n* = 6) from appropriate for gestational age (AGA) group (AUROC = 0.76) (140). And the expression of lncRNA RP11 (lncRNA RP11-290L1.3) from cord blood was positively correlated with birth weight (*r* = 0.8003, *P* < 0.01) (141). In addition, ncRNAs have multiple roles in the human placenta which connects mother and fetus, such as regulating trophoblast proliferation and differentiation, and affecting insulin secretion and regulation [reviewed in Žarković et al. (142)]. Thus, ncRNA expression may predict the fetal birth weight and metabolic risks in later life (Tables 2, 3).

Thamotharan et al. (143) found that inhibition of miR-10b could regulate the decrease in *E*-cadherin *in vitro*, and assumed that the increase in miR-10b in IUGR placentas could upregulate *E*-cadherin, which have a crucial role in development and tissue morphogenesis. They also found that miR-363 was increased in IUGR placentas, and miR-363 only responded to nutrition restriction *in vitro*, miR-363 downregulates sodium coupled neutral amino acid transporters (SNAT1 and SNAT2), thus reducing system amino acid transport activity which affected the fetal development (143). Zou et al. found that the expression level of miR-424 was significantly increased in IUGR placental tissues. They proposed that miR-424 might regulate estrogen-related receptor γ (ERRγ) and 17 beta-hydroxysteroid dehydrogenase type 1 (HSD17B1) modulating trophoblast-derived cell line proliferation and invasion to participate the pathogenesis of IUGR (144).

Some miRNAs from the chromosome 19 miRNA cluster (C19MC) are exclusively or abundantly expressed in the placenta, and these miRNAs in maternal plasma or serum samples may have diagnostic potential for the later occurrence of pregnancy-related complications such as IUGR (145). Higashijima et al. found that seven placenta-specific microRNAs from C19MC (miR-518b, miR-1323, miR-516b, miR-515-5p, miR-520h, miR-519d, and miR-526b) were decreased in placentas of human IUGR pregnancies (146). However, Jing et al. identified 5 C19MC miRNAs (miR-516a-5p, miR-516b-5p, miR-520a-3p, miR-1323, and miR-523-5p) that were upregulated in the fetal cord blood of obese mothers (147). For unclear reasons, it is possible that placental miRNA traffic primarily to the maternal circulation so have importantly different detection times in different nutritional models. Additionally, Wang et al. (148) found decreased expression of miR-518b and proposed that it could regulate placental trophoblast cells, thus contributing to IUGR and low fetal birth weight. Therefore, it is important to determine the specific pathways in which miR-518b is involved and how miR-518b contributes to IUGR in future study.

Maccani et al. found that miR-21 expression was reduced in placentas of SGA (49), and they validated PTEN (phosphatase and tensin homolog deleted on chromosome 10) as a target of miR-21 consistent with previous studies that investigated cancer. PTEN can regulate glucose metabolism through the PI3K/AKT pathway [reviewed in Chen et al. (149)] and directly suppress glycolysis by dephosphorylation and inhibition of phosphoglycerate kinase 1 (PGK1) (150). Therefore, these findings suggest that the miR-21-PTEN axis might be involved in poor fetal growth and future diseases. Interestingly, the expression of miR-21 was increased in placentas of macrosomia (115, 151). miR-21 might target the MAPK signaling pathway, PI3K/AKT signaling pathway, and mTOR signaling pathway (Table 2) (151) to increase the risk of macrosomia and metabolic diseases in the future. Therefore, the detection of miR-21 expression in the placenta may predict the risk of later metabolic diseases and also be a potential therapeutic and diagnostic method.

miR-141, belonging to the miR-200 family, can regulate insulin-like growth factor 2 (IGF2), thus participating in fetal and placental development in mice (152). Tang et al. (153) found an increase of miR-141 was associated with a decrease in pleomorphic adenoma gene 1 (PLAG1) and IGF2 in the placenta of IUGR patients according to correlation analysis. PLAG1, a growth regulator (154), is known to target IGF2 in some tumors (155–157). IGF2 is critical for early human placental development (153), prenatal growth (158), and metabolism (159). In addition, the overexpression of IGF2 is involved in somatic overgrowth. Consistent with these studies, the miR-141-PLAG1-IGF2 network might exert an action on IUGR and even metabolic disorders later in life (153).

miR-16 was markedly reduced in infants with LBW (*P* < 0.05) and could well predict the risk of SGA status (*P* = 0.009) (49). In contrast, miR-16 did not change in placenta of macrosomia (Table 2) (115, 151). For unclear reasons, this difference may be linked to the expression of its known target BCL-2, an inhibitor of apoptosis (160). In addition to miR-16, the expression of miR-519 was also different in newborns with different birth weights. miR-519 can modulate its target to strongly inhibit cell proliferation (161) and growth (162). In placentas of IUGR, miR-519a was upregulated and might regulate placental trophoblast function via its putative targets Grb2-associated binder 1 (Gab1), PTEN, and hypoxia-inducible transcription factors 1α (HIF-1α), thus participating in the pathogenesis of LBW (148).

Of note, in 2020, instead of investigating the LBW groups, Wang et al. (163) studied miRNAs in the placenta of macrosomia, finding that circRNA-SETD2 (hsa-circRNA-103345) is upregulated. Compared with miRNAs, the mechanism of circRNAs is not fully understood and there are some controversies. However, circRNAs are mainly considered as miRNA inhibitors (or “sponges”) (164), and then regulate gene expression at post-transcriptional levels. So they explored the downstream target of circRNA-SETD2 in cell experiment, verified that the circRNA-SETD2/miR-519a/PTEN axis (163). And miR-519a has been mentioned previously to be involved in regulating birth weight. So the potential mechanisms of up-regulating circRNA-SETD2 in macrosomia is to inhibit miR-519a causing the increased expression of PTEN, then increasing the risks of metabolic diseases.

Different from miRNA mechanisms, some lncRNAs competitively target miRNAs, thereby attenuating the degradation or inhibition of miRNAs, then regulating downstream protein-coding target genes (165). This is similar to the mechanism of some circRNAs acting as competitive endogenous RNA (ceRNA). lncRNA RP11 was found to control adipocyte differentiation in visceral adipose tissue (166). And it is preferentially expressed in subcutaneous/visceral adipose tissue according to genome-wide association studies (GWAS) (167). lncRNA RP11 was increased significantly in the umbilical cord blood of GDM-induced macrosomia, then regulating target genes such as PPARγ, sterol regulatory element-binding protein 1C (SREBP-1c), and FASN, which might be the reason for fetal fat accumulation in GDM (141).

lncRNA H19 gene was expressed abundantly in the placenta and recent studies showed that its expression was reduced in IUGR placentas compared to healthy placental controls, suggesting that it regulates IUGR (168, 169). The underlying mechanism is that lncRNA H19 regulates the PI3K/AKT pathway, disrupts trophoblast cell function (170), and promotes autophagy by targeting miR-18a-5p (Table 3) (169). Additionally, autophagy is magnified in IUGR by the reduction of lncRNA H19 (169).

## Future prospects

Prenatal nutrition plays a critical role in shaping the road of health and disease later in life. There are not many studies regarding ncRNA dysregulation as well as its role in offspring exposed to different maternal nutrition. However, it is clear that existing studies have found significant differences in expression and action, so we turned to the possibility of using miRNAs as early diagnostic tools and therapeutic targets.

The sampling site of ncRNAs is particularly important. In this review, we mainly discussed ncRNAs derived from liver, fat, pancreas, skeletal muscle, and bone marrow of animal offspring and placenta, umbilical cord blood and dried blood spots of human newborns. Of note, studies of ncRNAs derived from maternal and fetal blood is in small amount. Also, some sources of ncRNAs such as breast milk, are not covered. But ncRNAs in human breast milk also affect the health of newborns (171). So this is an area that can be pursued in the future. In addition, in clinical practice, placenta and cord blood is relatively easy to obtain and ethics committee approval compared with fetal liver, pancreas etc. And alternations of placental miRNAs expression may serve as a record of intrauterine exposure (172). Therefore, placenta is a excellent type of sample to study how maternal nutrition affect fetus.

In addition, the time of sample collection is worth mentioning. Since the offspring metabolism, such as impaired glucose tolerance or insulin resistance, shows varying degrees of impairment in infancy, adulthood, and older age. Similarly, the expression of ncRNAs is not static. So it is important to find the cut-off time for changes in glucose and lipid metabolism or ncRNAs expression so that we can detect metabolic disease at an early stage and respond to it.

Some miRNAs have been shown to be, or have great potential to be, early warning indicators of obesity, T2D, or NAFLD changes. First, serum miR-122 showed the great diagnostic accuracy for NAFLD (AUROC ≥ 0.85) (62). There have also been attempts to discriminate SGA and AGA groups using serum miRNAs associated with metabolic alterations (such as miR-122), but there were no positive results (173). The levels of hepatic miR-122 in the offspring exposed to maternal malnutrition were reduced, as we noted earlier. Although the detection time points were different, we can speculate that miR-122 is critical. Perhaps before malnourished offspring had time to exhibit a catch-up growth phenotype, miRNAs have changed toward the direction of overnutrition due to a mismatch between prenatal and postnatal environments. Serum miR-15b can be used to identify T2D patients (AUROC = 0.969) (114). miR-141 in the placenta could serve as a potential biomarker to distinguish IUGR from normal controls, with an area under the ROC curve of 0.839, a sensitivity of 88.5%, and a specificity of 71.7% (153).

miRNAs can also be potential therapeutic targets. At first, anti-miR induced let-7 knockdown (133) and let-7 anti-miR transfection (26) are possible treatments for T2D and NAFLD, respectively. Next, the loss of miR-342 can protect against obesity and diabetes (111). Finally, a maternal diet enriched in olive oil may prevent the reduced expression of miR-122 in fetuses of GDM mothers, thus preventing the abnormal liver lipid metabolism (78, 81).

We concluded that the expression levels of miR-21, miR-370, miR-16, and miR-143 in offspring are related to maternal nutritional models. However, hepatic miR-122 expression in offspring decreased regardless of the nutritional status of the mother. Of note, the patterns of some ncRNAs cannot be summarized due to too few studies. Again, these results indicated that ncRNA plays a broad role in mediating the effects of an adverse intrauterine environment on poor metabolic health in offspring.

The early life environment influences the risk of developing diseases such as the MetS. How the maternal nutrition status influences the health and disease of her offspring can be explained by epigenetics. However, the mechanisms by which developmental programming may be transmitted to further generations are unclear. We believe that the role of ncRNA in linking maternal nutritional status to offspring metabolism will be developed over time.

## Author contributions

YZ drafted the manuscript. YW, QZ, and XX revised the manuscript. All authors contributed to the article and approved the submitted version.

## References

[1] Muzurović E Mikhailidis DP Mantzoros C. Non-alcoholic fatty liver disease, insulin resistance, metabolic syndrome and their association with vascular risk. *Metabolism.* (2021) 119:154770. 10.1016/j.metabol.2021.154770 33864798

[2] Hirode G Wong RJ. Trends in the prevalence of metabolic syndrome in the United States, 2011-2016. *JAMA.* (2020) 323:2526–8. 10.1001/jama.2020.4501 32573660 PMC7312413

[3] Gutiérrez-Solis AL Datta Banik S Méndez-González RM. Prevalence of metabolic syndrome in mexico: a systematic review and meta-analysis. *Metab Syndr Relat Disord.* (2018) 16:395–405. 10.1089/met.2017.0157 30063173

[4] Eckel N Li Y Kuxhaus O Stefan N Hu FB Schulze MB. Transition from metabolic healthy to unhealthy phenotypes and association with cardiovascular disease risk across BMI categories in 90 257 women (the Nurses’ Health Study): 30 year follow-up from a prospective cohort study. *Lancet Diabetes Endocrinol.* (2018) 6:714–24. 10.1016/s2213-8587(18)30137-2 29859908

[5] Low Wang CC Hess CN Hiatt WR Goldfine AB. Clinical update: cardiovascular disease in diabetes mellitus: atherosclerotic cardiovascular disease and heart failure in type 2 diabetes mellitus – mechanisms, management, and clinical considerations. *Circulation.* (2016) 133:2459–502. 10.1161/circulationaha.116.022194 27297342 PMC4910510

[6] Targher G Byrne CD Tilg H. NAFLD and increased risk of cardiovascular disease: clinical associations, pathophysiological mechanisms and pharmacological implications. *Gut.* (2020) 69:1691–705. 10.1136/gutjnl-2020-320622 32321858

[7] Virani SS Alonso A Aparicio HJ Benjamin EJ Bittencourt MS Callaway CW Heart disease and stroke statistics-2021 update: a report from the American Heart Association. *Circulation.* (2021) 143:e254–743. 10.1161/cir.0000000000000950 33501848 PMC13036842

[8] Author. On the Front Line: Obesity and NAFLD. *Cell Metab.* (2020) 31:655–7. 10.1016/j.cmet.2020.03.014 32268108

[9] Ling C Rönn T. Epigenetics in human obesity and type 2 diabetes. *Cell Metab.* (2019) 29:1028–44. 10.1016/j.cmet.2019.03.009 30982733 PMC6509280

[10] Tilg H Moschen AR Roden M. NAFLD and diabetes mellitus. *Nat Rev Gastroenterol Hepatol.* (2017) 14:32–42. 10.1038/nrgastro.2016.147 27729660

[11] Stefan N Cusi K. A global view of the interplay between non-alcoholic fatty liver disease and diabetes. *Lancet Diabetes Endocrinol.* (2022) 10:284–96. 10.1016/s2213-8587(22)00003-1 35183303

[12] Ferguson D Finck BN. Emerging therapeutic approaches for the treatment of NAFLD and type 2 diabetes mellitus. *Nat Rev Endocrinol.* (2021) 17:484–95. 10.1038/s41574-021-00507-z 34131333 PMC8570106

[13] Barnett TA Kelly AS Young DR Perry CK Pratt CA Edwards NM Sedentary behaviors in today’s youth: approaches to the prevention and management of childhood obesity: a scientific statement from the American Heart Association. *Circulation.* (2018) 138:e142–59. 10.1161/cir.0000000000000591 30354382

[14] Hills AP Arena R Khunti K Yajnik CS Jayawardena R Henry CJ Epidemiology and determinants of type 2 diabetes in South Asia. *Lancet Diabetes Endocrinol.* (2018) 6:966–78. 10.1016/s2213-8587(18)30204-3 30287102

[15] Marchesini G Petta S Dalle Grave R. Diet, weight loss, and liver health in nonalcoholic fatty liver disease: Pathophysiology, evidence, and practice. *Hepatology.* (2016) 63:2032–43. 10.1002/hep.28392 26663351

[16] Querter I Pauwels NS De Bruyne R Dupont E Verhelst X Devisscher L Maternal and perinatal risk factors for pediatric nonalcoholic fatty liver disease: a systematic review. *Clin Gastroenterol Hepatol.* (2022) 20:740–55. 10.1016/j.cgh.2021.04.014 33862225

[17] Vaiserman A Lushchak O. Developmental origins of type 2 diabetes: Focus on epigenetics. *Ageing Res Rev.* (2019) 55:100957. 10.1016/j.arr.2019.100957 31473332

[18] Barker DJ Osmond C. Infant mortality, childhood nutrition, and ischaemic heart disease in England and Wales. *Lancet.* (1986) 1:1077–81. 2871345 10.1016/s0140-6736(86)91340-1

[19] Ravelli AC van der Meulen JH Michels RP Osmond C Barker DJ Hales CN Glucose tolerance in adults after prenatal exposure to famine. *Lancet.* (1998) 351:173–7. 10.1016/s0140-6736(97)07244-9 9449872

[20] Ravelli GP Stein ZA Susser MW. Obesity in young men after famine exposure in utero and early infancy. *New Engl J Med.* (1976) 295:349–53. 10.1056/nejm197608122950701 934222

[21] Li C Lumey LH. Exposure to the Chinese famine of 1959-61 in early life and long-term health conditions: a systematic review and meta-analysis. *Int J Epidemiol.* (2017) 46:1157–70. 10.1093/ije/dyx013 28338900

[22] Zou Z Li C Patton GC. Early-life exposure to the Chinese Famine and subsequent T2DM. *Nat Rev Endocrinol.* (2020) 16:124–5. 10.1038/s41574-019-0299-y 31784714

[23] Wang J Li Y Han X Liu B Hu H Wang F Exposure to the Chinese famine in childhood increases type 2 diabetes risk in adults. *J Nutr.* (2016) 146:2289–95. 10.3945/jn.116.234575 27629572

[24] Zhu Y Olsen SF Mendola P Halldorsson TI Rawal S Hinkle SN Maternal consumption of artificially sweetened beverages during pregnancy, and offspring growth through 7 years of age: a prospective cohort study. *Int J Epidemiol.* (2017) 46:1499–508. 10.1093/ije/dyx095 28586472 PMC5837735

[25] Sinzato YK Paula VG Gallego FQ Moraes-Souza RQ Corrente JE Volpato GT Maternal diabetes and postnatal high-fat diet on pregnant offspring. *Front Cell Dev Biol.* (2022) 10:818621. 10.3389/fcell.2022.818621 35706903 PMC9189289

[26] Simino LAP Panzarin C Fontana MF de Fante T Geraldo MV Ignácio-Souza LM MicroRNA Let-7 targets AMPK and impairs hepatic lipid metabolism in offspring of maternal obese pregnancies. *Sci Rep.* (2021) 11:8980. 10.1038/s41598-021-88518-8 33903707 PMC8076304

[27] Zhou LY Deng MQ Zhang Q Xiao XH. Early-life nutrition and metabolic disorders in later life: a new perspective on energy metabolism. *Chin Med J.* (2020) 133:1961–70. 10.1097/cm9.0000000000000976 32826460 PMC7462214

[28] Boehmer BH Limesand SW Rozance PJ. The impact of IUGR on pancreatic islet development and beta-cell function. *J Endocrinol.* (2017) 235:R63–76. 10.1530/JOE-17-0076 28808079 PMC5808569

[29] Fitz-James MH Cavalli G. Molecular mechanisms of transgenerational epigenetic inheritance. *Nat Rev Genet.* (2022) [Epub ahead of print]. 10.1038/s41576-021-00438-5 34983971 PMC7619059

[30] Miska EA Ferguson-Smith AC. Transgenerational inheritance: Models and mechanisms of non-DNA sequence-based inheritance. *Science.* (2016) 354:59–63. 10.1126/science.aaf4945 27846492

[31] ENCODE Project Consortium. An integrated encyclopedia of DNA elements in the human genome. *Nature.* (2012) 489:57–74. 10.1038/nature11247 22955616 PMC3439153

[32] Anastasiadou E Jacob LS Slack FJ. Non-coding RNA networks in cancer. *Nature Rev Cancer.* (2018) 18:5–18. 10.1038/nrc.2017.99 29170536 PMC6337726

[33] Poller W Dimmeler S Heymans S Zeller T Haas J Karakas M Non-coding RNAs in cardiovascular diseases: diagnostic and therapeutic perspectives. *Eur Heart J.* (2018) 39:2704–16. 10.1093/eurheartj/ehx165 28430919 PMC6454570

[34] Wei LH Guo JU. Coding functions of “noncoding”, RNAs. *Science.* (2020) 367:1074–5. 10.1126/science.aba6117 32139529

[35] Beermann J Piccoli MT Viereck J Thum T. Non-coding RNAs in development and disease: background, mechanisms, and therapeutic approaches. *Physiol Rev.* (2016) 96:1297–325. 10.1152/physrev.00041.2015 27535639

[36] Saeedi Borujeni MJ Esfandiary E Baradaran A Valiani A Ghanadian M Codoñer-Franch P Molecular aspects of pancreatic β-cell dysfunction: oxidative stress, microRNA, and long noncoding RNA. *J Cell Physiol.* (2019) 234:8411–25. 10.1002/jcp.27755 30565679

[37] Bartel DP. MicroRNAs: genomics, biogenesis, mechanism, and function. *Cell.* (2004) 116:281–97. 10.1016/s0092-8674(04)00045-5 14744438

[38] Kristensen LS Andersen MS Stagsted LVW Ebbesen KK Hansen TB Kjems J. The biogenesis, biology and characterization of circular RNAs. *Nat Rev Genet.* (2019) 20:675–91. 10.1038/s41576-019-0158-7 31395983

[39] Kopp F Mendell JT. Functional classification and experimental dissection of long noncoding RNAs. *Cell.* (2018) 172:393–407. 10.1016/j.cell.2018.01.011 29373828 PMC5978744

[40] Chen LL. The expanding regulatory mechanisms and cellular functions of circular RNAs. *Nat Rev Mol Cell Biol.* (2020) 21:475–90. 10.1038/s41580-020-0243-y 32366901

[41] Floris I Kraft JD Altosaar I. Roles of MicroRNA across prenatal and postnatal periods. *Int J Mol Sci.* (2016) 17:1994. 10.3390/ijms17121994 27916805 PMC5187794

[42] Agbu P Carthew RW. MicroRNA-mediated regulation of glucose and lipid metabolism. *Nat Rev Mol Cell Biol.* (2021) 22:425–38. 10.1038/s41580-021-00354-w 33772227 PMC8853826

[43] Repiská G Koneèná B Shelke GV Lässer C Vlková BI Minárik G. Is the DNA of placental origin packaged in exosomes isolated from plasma and serum of pregnant women? *Clin Chem Lab Med.* (2018) 56:e150–3. 10.1515/cclm-2017-0560 29306910

[44] Kim SH MacIntyre DA Binkhamis R Cook J Sykes L Bennett PR Maternal plasma miRNAs as potential biomarkers for detecting risk of small-for-gestational-age births. *EBioMedicine.* (2020) 62:103145. 10.1016/j.ebiom.2020.103145 33260001 PMC7708817

[45] Rodosthenous RS Burris HH Sanders AP Just AC Dereix AE Svensson K Second trimester extracellular microRNAs in maternal blood and fetal growth: An exploratory study. *Epigenetics.* (2017) 12:804–10. 10.1080/15592294.2017.1358345 28758828 PMC5739092

[46] Maccani MA Marsit CJ. Epigenetics in the placenta. *Am J Reprod Immunol.* (2009) 62:78–89. 10.1111/j.1600-0897.2009.00716.x 19614624 PMC2813777

[47] Barad O Meiri E Avniel A Aharonov R Barzilai A Bentwich I MicroRNA expression detected by oligonucleotide microarrays: system establishment and expression profiling in human tissues. *Genome Res.* (2004) 14:2486–94. 10.1101/gr.2845604 15574827 PMC534673

[48] Chang G Mouillet JF Mishima T Chu T Sadovsky E Coyne CB Expression and trafficking of placental microRNAs at the feto-maternal interface. *FASEB J.* (2017) 31:2760–70. 10.1096/fj.201601146R 28289056 PMC5471515

[49] Maccani MA Padbury JF Marsit CJ. miR-16 and miR-21 expression in the placenta is associated with fetal growth. *PLoS One.* (2011) 6:e21210. 10.1371/journal.pone.0021210 21698265 PMC3115987

[50] Lagos-Quintana M Rauhut R Yalcin A Meyer J Lendeckel W Tuschl T. Identification of tissue-specific microRNAs from mouse. *Curr Biol.* (2002) 12:735–9. 10.1016/s0960-9822(02)00809-6 12007417

[51] Chang J Nicolas E Marks D Sander C Lerro A Buendia MA miR-122, a mammalian liver-specific microRNA, is processed from hcr mRNA and may downregulate the high affinity cationic amino acid transporter CAT-1. *RNA Biol.* (2004) 1:106–13. 10.4161/rna.1.2.1066 17179747

[52] Mishra S Rizvi A Pradhan A Perrone MA Ali W. Circulating microRNA-126 &122 in patients with coronary artery disease: correlation with small dense LDL. *Prostagland Other Lipid Mediat.* (2021) 153:106536. 10.1016/j.prostaglandins.2021.106536 33556577

[53] Refeat MM Hassan NA Ahmad IH Mostafa ERM Amr KS. Correlation of circulating miRNA-33a and miRNA-122 with lipid metabolism among Egyptian patients with metabolic syndrome. *J Genetic Eng Biotechnol.* (2021) 19:147. 10.1186/s43141-021-00246-8 34611771 PMC8492848

[54] Bandiera S Pfeffer S Baumert TF Zeisel MB. miR-122–a key factor and therapeutic target in liver disease. *J Hepatol.* (2015) 62:448–57. 10.1016/j.jhep.2014.10.004 25308172

[55] Thakral S Ghoshal K. miR-122 is a unique molecule with great potential in diagnosis, prognosis of liver disease, and therapy both as miRNA mimic and antimir. *Curr Gene Therapy.* (2015) 15:142–50. 10.2174/1566523214666141224095610 25537773 PMC4439190

[56] Satishchandran A Ambade A Rao S Hsueh YC Iracheta-Vellve A Tornai D MicroRNA 122, Regulated by GRLH2, protects livers of mice and patients from ethanol-induced liver disease. *Gastroenterology.* (2018) 154:238–52.e7. 10.1053/j.gastro.2017.09.022 28987423 PMC5742049

[57] Long JK Dai W Zheng YW Zhao SP. miR-122 promotes hepatic lipogenesis via inhibiting the LKB1/AMPK pathway by targeting Sirt1 in non-alcoholic fatty liver disease. *Mol Med.* (2019) 25:26. 10.1186/s10020-019-0085-2 31195981 PMC6567918

[58] Chung HH. New insights for controversial issues of miR-122 in hepatic lipid metabolism. *Gastroenterology.* (2018) 154:1552–3. 10.1053/j.gastro.2017.12.039 29526733

[59] González-Arce LM Lara-Riegos JC Pérez-Mendoza GJ Rubí-Castellanos R Vega-Marcín M Valencia-Pacheco G High expression levels of circulating microRNA-122 and microRNA-222 are associated with obesity in children with Mayan ethnicity. *Am J Hum Biol.* (2021) 33:e23540. 10.1002/ajhb.23540 33226155

[60] Mohany KM Al Rugaie O Al-Wutayd O Al-Nafeesah A. Investigation of the levels of circulating miR-29a, miR-122, sestrin 2 and inflammatory markers in obese children with/without type 2 diabetes: a case control study. *BMC Endocr Disord.* (2021) 21:152. 10.1186/s12902-021-00829-z 34344352 PMC8330040

[61] Hess AL Larsen LH Udesen PB Sanz Y Larsen TM Dalgaard LT. Levels of circulating miR-122 are associated with weight loss and metabolic syndrome. *Obesity.* (2020) 28:493–501. 10.1002/oby.22704 32090516

[62] Salvoza NC Klinzing DC Gopez-Cervantes J Baclig MO. Association of circulating serum miR-34a and miR-122 with dyslipidemia among patients with non-alcoholic fatty liver disease. *PLoS One.* (2016) 11:e0153497. 10.1371/journal.pone.0153497 27077736 PMC4831793

[63] Pillai SS Lakhani HV Zehra M Wang J Dilip A Puri N Predicting nonalcoholic fatty liver disease through a panel of plasma biomarkers and microRNAS in female West Virginia Population. *Int J Mol Sci.* (2020) 21:6698. 10.3390/ijms21186698 32933141 PMC7554851

[64] Zhang X Asllanaj E Amiri M Portilla-Fernandez E Bramer WM Nano J Deciphering the role of epigenetic modifications in fatty liver disease: a systematic review. *Eur J Clin Investig.* (2021) 51:e13479. 10.1111/eci.13479 33350463 PMC8243926

[65] Zheng J Xiao X Zhang Q Wang T Yu M Xu J. Maternal low-protein diet modulates glucose metabolism and hepatic MicroRNAs expression in the early life of offspring †. *Nutrients.* (2017) 9:205. 10.3390/nu9030205 28264458 PMC5372868

[66] Benatti RO Melo AM Borges FO Ignacio-Souza LM Simino LA Milanski M Maternal high-fat diet consumption modulates hepatic lipid metabolism and microRNA-122 (miR-122) and microRNA-370 (miR-370) expression in offspring. *Br J Nutr.* (2014) 111:2112–22. 10.1017/s0007114514000579 24666709

[67] Chai C Rivkin M Berkovits L Simerzin A Zorde-Khvalevsky E Rosenberg N Metabolic Circuit Involving Free Fatty Acids, microRNA 122, and Triglyceride Synthesis in Liver and Muscle Tissues. *Gastroenterology.* (2017) 153:1404–15. 10.1053/j.gastro.2017.08.013 28802563

[68] Wen J Friedman JR. miR-122 regulates hepatic lipid metabolism and tumor suppression. *J Clin Investig.* (2012) 122:2773–6. 10.1172/jci63966 22820284 PMC3408753

[69] Qiang J Tao YF Bao JW Chen J Li HX He J High fat diet-induced miR-122 regulates lipid metabolism and fat deposition in genetically improved farmed tilapia (GIFT, Oreochromis niloticus) Liver. *Front Physiol.* (2018) 9:1422. 10.3389/fphys.2018.01422 30344495 PMC6182080

[70] Hodson L Fielding BA. Stearoyl-CoA desaturase: rogue or innocent bystander? *Progress Lipid Res.* (2013) 52:15–42. 10.1016/j.plipres.2012.08.002 23000367

[71] Agarwal AK Tunison K Dalal JS Nagamma SS Hamra FK Sankella S Metabolic, reproductive, and neurologic abnormalities in agpat1-null mice. *Endocrinology.* (2017) 158:3954–73. 10.1210/en.2017-00511 28973305 PMC5695831

[72] Cao B Liu C Zhang Q Dong Y. Maternal high-fat diet leads to non-alcoholic fatty liver disease through upregulating hepatic SCD1 Expression in Neonate Rats. *Front Nutr.* (2020) 7:581723. 10.3389/fnut.2020.581723 33282902 PMC7705221

[73] Dai Y Ghosh S Shin BC Devaskar SU. Role of microRNA-122 in hepatic lipid metabolism of the weanling female rat offspring exposed to prenatal and postnatal caloric restriction. *J Nutr Biochem.* (2019) 73:108220. 10.1016/j.jnutbio.2019.108220 31630081 PMC6896790

[74] Norman KL Sarnow P. Modulation of hepatitis C virus RNA abundance and the isoprenoid biosynthesis pathway by microRNA miR-122 involves distinct mechanisms. *J Virol.* (2010) 84:666–70. 10.1128/jvi.01156-09 19846523 PMC2798415

[75] Jiang G Li Z Liu F Ellsworth K Dallas-Yang Q Wu M Prevention of obesity in mice by antisense oligonucleotide inhibitors of stearoyl-CoA desaturase-1. *J Clin Investig.* (2005) 115:1030–8. 10.1172/jci23962 15761499 PMC1062893

[76] Aiken CE Ozanne SE. Transgenerational developmental programming. *Hum Reprod Update.* (2014) 20:63–75. 10.1093/humupd/dmt043 24082037

[77] Lane M Robker RL Robertson SA. Parenting from before conception. *Science.* (2014) 345:756–60. 10.1126/science.1254400 25124428

[78] Fornes D Heinecke F Roberti SL White V Capobianco E Jawerbaum A. Proinflammation in maternal and fetal livers and circulating miR-122 dysregulation in a GDM rat model induced by intrauterine programming. *Mol Cell Endocrinol.* (2020) 510:110824. 10.1016/j.mce.2020.110824 32315718

[79] Fornes D White V Higa R Heinecke F Capobianco E Jawerbaum A. Sex-dependent changes in lipid metabolism, PPAR pathways and microRNAs that target PPARs in the fetal liver of rats with gestational diabetes. *Mol Cell Endocrinol.* (2018) 461:12–21. 10.1016/j.mce.2017.08.004 28807878

[80] Capobianco E Gomez Ribot D Fornes D Powell TL Levieux C Jansson T Diet enriched with olive oil attenuates placental dysfunction in rats with gestational diabetes induced by intrauterine programming. *Mol Nutr Food Res.* (2018) 62:e1800263. 10.1002/mnfr.201800263 29939470

[81] Fornes D Gomez Ribot D Heinecke F Roberti SL Capobianco E Jawerbaum A. Maternal diets enriched in olive oil regulate lipid metabolism and levels of PPARs and their coactivators in the fetal liver in a rat model of gestational diabetes mellitus. *J Nutr Biochem.* (2020) 78:108334. 10.1016/j.jnutbio.2019.108334 32004928

[82] Zheng J Zhang Q Mul JD Yu M Xu J Qi C Maternal high-calorie diet is associated with altered hepatic microRNA expression and impaired metabolic health in offspring at weaning age. *Endocrine.* (2016) 54:70–80. 10.1007/s12020-016-0959-9 27106801

[83] Ciavarella C Motta I Vasuri F Fittipaldi S Valente S Pollutri D Involvement of miR-30a-5p and miR-30d in endothelial to mesenchymal transition and early osteogenic commitment under inflammatory Stress in HUVEC. *Biomolecules.* (2021) 11:226. 10.3390/biom11020226 33562690 PMC7915105

[84] Zaragosi LE Wdziekonski B Brigand KL Villageois P Mari B Waldmann R Small RNA sequencing reveals miR-642a-3p as a novel adipocyte-specific microRNA and miR-30 as a key regulator of human adipogenesis. *Genome Biol.* (2011) 12:R64. 10.1186/gb-2011-12-7-r64 21767385 PMC3218826

[85] Nunez Lopez YO Garufi G Pasarica M Seyhan AA. Elevated and correlated expressions of miR-24, miR-30d, miR-146a, and SFRP-4 in human abdominal adipose tissue play a role in adiposity and insulin resistance. *Int J Endocrinol.* (2018) 2018:7351902. 10.1155/2018/7351902 29721017 PMC5867542

[86] Gong M Antony S Sakurai R Liu J Iacovino M Rehan VK. Bone marrow mesenchymal stem cells of the intrauterine growth-restricted rat offspring exhibit enhanced adipogenic phenotype. *Int J Obesity.* (2016) 40:1768–75. 10.1038/ijo.2016.157 27599633 PMC5113998

[87] Zhu W Gui W Lin X Yin X Liang L Li H. Maternal undernutrition modulates hepatic MicroRNAs expression in the early life of offspring. *Exp Cell Res.* (2021) 400:112450. 10.1016/j.yexcr.2020.112450 33347859

[88] Sosa-Larios TC Miliar-Garcia A Reyes-Castro LA Morimoto S Jaramillo-Flores ME. Alterations in lipid metabolism due to a protein-restricted diet in rats during gestation and/or lactation. *Food Funct.* (2017) [Epub ahead of print]. 10.1039/c7fo01513e 29099131

[89] Nguyen LT Chen H Zaky A Pollock C Saad S. SIRT1 overexpression attenuates offspring metabolic and liver disorders as a result of maternal high-fat feeding. *J Physiol.* (2019) 597:467–80. 10.1113/jp276957 30381838 PMC6332732

[90] Yamazaki M Munetsuna E Yamada H Ando Y Mizuno G Fujii R Maternal fructose consumption down-regulates Lxra expression via miR-206-mediated regulation. *J Nutr Biochem.* (2020) 82:108386. 10.1016/j.jnutbio.2020.108386 32388164

[91] Zhong D Huang G Zhang Y Zeng Y Xu Z Zhao Y MicroRNA-1 and microRNA-206 suppress LXRα-induced lipogenesis in hepatocytes. *Cell Signal.* (2013) 25:1429–37. 10.1016/j.cellsig.2013.03.003 23499676

[92] Wu H Zhang T Pan F Steer CJ Li Z Chen X MicroRNA-206 prevents hepatosteatosis and hyperglycemia by facilitating insulin signaling and impairing lipogenesis. *J Hepatol.* (2017) 66:816–24. 10.1016/j.jhep.2016.12.016 28025059 PMC5568011

[93] Martinez D Pentinat T Ribo S Daviaud C Bloks VW Cebria J In utero undernutrition in male mice programs liver lipid metabolism in the second-generation offspring involving altered Lxra DNA methylation. *Cell Metab.* (2014) 19:941–51. 10.1016/j.cmet.2014.03.026 24794974

[94] Personnaz J Piccolo E Dortignac A Iacovoni JS Mariette J Rocher V Nuclear HMGB1 protects from nonalcoholic fatty liver disease through negative regulation of liver X receptor. *Sci Adv.* (2022) 8:eabg9055. 10.1126/sciadv.abg9055 35333579 PMC8956270

[95] Iliopoulos D Drosatos K Hiyama Y Goldberg IJ Zannis VI. MicroRNA-370 controls the expression of microRNA-122 and Cpt1alpha and affects lipid metabolism. *J Lipid Res.* (2010) 51:1513–23. 10.1194/jlr.M004812 20124555 PMC3035515

[96] Panzarin C Simino LAP Mancini MCS Ignácio-Souza LM Milanski M Torsoni MA Hepatic microRNA modulation might be an early event to non-alcoholic fatty liver disease development driven by high-fat diet in male mice. *Mol Biol Rep.* (2022) 49:2655–66. 10.1007/s11033-021-07072-8 35048271

[97] Ortega FJ Mercader JM Moreno-Navarrete JM Rovira O Guerra E Esteve E Profiling of circulating microRNAs reveals common microRNAs linked to type 2 diabetes that change with insulin sensitization. *Diabetes Care.* (2014) 37:1375–83. 10.2337/dc13-1847 24478399

[98] Zampetaki A Kiechl S Drozdov I Willeit P Mayr U Prokopi M Plasma microRNA profiling reveals loss of endothelial miR-126 and other microRNAs in type 2 diabetes. *Circ Res.* (2010) 107:810–7. 10.1161/circresaha.110.226357 20651284

[99] Witkowski M Weithauser A Tabaraie T Steffens D Kränkel N Witkowski M Micro-RNA-126 reduces the blood thrombogenicity in diabetes mellitus via targeting of tissue factor. *Arterioscl Thromb Vasc Biol.* (2016) 36:1263–71. 10.1161/atvbaha.115.306094 27127202 PMC4894779

[100] Rawal S Munasinghe PE Shindikar A Paulin J Cameron V Manning P Down-regulation of proangiogenic microRNA-126 and microRNA-132 are early modulators of diabetic cardiac microangiopathy. *Cardiovasc Res.* (2017) 113:90–101. 10.1093/cvr/cvw235 28065883

[101] Pishavar E Behravan J. miR-126 as a therapeutic agent for diabetes mellitus. *Curr Pharm Design.* (2017) 23:3309–14. 10.2174/1381612823666170424120121 28440196

[102] Ortiz-Dosal A Arellanes-Licea EDC Rodil-García P Salazar-Olivo LA. Circulating microRNAs overexpressed in macrosomia: an experimental and bioinformatic approach. *J Dev Origins Health Dis.* (2020) 11:464–72. 10.1017/s2040174420000422 32452339

[103] Fernandez-Twinn DS Alfaradhi MZ Martin-Gronert MS Duque-Guimaraes DE Piekarz A Ferland-McCollough D Downregulation of IRS-1 in adipose tissue of offspring of obese mice is programmed cell-autonomously through post-transcriptional mechanisms. *Mol Metab.* (2014) 3:325–33. 10.1016/j.molmet.2014.01.007 24749062 PMC3986586

[104] de Almeida-Faria J Duque-Guimarães DE Ong TP Pantaleão LC Carpenter AA Loche E Maternal obesity during pregnancy leads to adipose tissue ER stress in mice via miR-126-mediated reduction in Lunapark. *Diabetologia.* (2021) 64:890–902. 10.1007/s00125-020-05357-4 33501603 PMC7940301

[105] Chen S Desai T McNew JA Gerard P Novick PJ Ferro-Novick S. Lunapark stabilizes nascent three-way junctions in the endoplasmic reticulum. *Proc Natl Acad Sci U.S.A.* (2015) 112:418–23. 10.1073/pnas.1423026112 25548161 PMC4299238

[106] de Almeida Faria J Duque-Guimarães D Carpenter AA Loche E Ozanne SE. A post-weaning obesogenic diet exacerbates the detrimental effects of maternal obesity on offspring insulin signaling in adipose tissue. *Sci Rep.* (2017) 7:44949. 10.1038/srep44949 28338072 PMC5364470

[107] Školníková E Šedová L Chylíková B Kábelová A Liška F Šeda O. Maternal high-sucrose diet affects phenotype outcome in adult male offspring: role of Zbtb16. *Front Genetics.* (2020) 11:529421. 10.3389/fgene.2020.529421 33061941 PMC7518089

[108] Alejandro EU Gregg B Wallen T Kumusoglu D Meister D Chen A Maternal diet-induced microRNAs and mTOR underlie β cell dysfunction in offspring. *J Clin Investig.* (2014) 124:4395–410. 10.1172/jci74237 25180600 PMC4191023

[109] Alejandro EU Jo S Akhaphong B Llacer PR Gianchandani M Gregg B Maternal low-protein diet on the last week of pregnancy contributes to insulin resistance and β-cell dysfunction in the mouse offspring. *Am J Physiol Regul Integr Comp Physiol.* (2020) 319:R485–96. 10.1152/ajpregu.00284.2019 32877242 PMC7717124

[110] Kaviani M Azarpira N Karimi MH Al-Abdullah I. The role of microRNAs in islet β-cell development. *Cell Biol Int.* (2016) 40:1248–55. 10.1002/cbin.10691 27743454

[111] Zhang D Yamaguchi S Zhang X Yang B Kurooka N Sugawara R Upregulation of Mir342 in diet-induced obesity mouse and the hypothalamic appetite control. *Front Endocrinol.* (2021) 12:727915. 10.3389/fendo.2021.727915 34526970 PMC8437242

[112] Li Y Chen Y Liu Z Lin B Deng X Xiao Q Downregulation of Kcnq1ot1 attenuates β-cell proliferation and insulin secretion via the miR-15b-5p/Ccnd1 and Ccnd2 axis. *Acta Diabetol.* (2022) [Epub ahead of print]. 10.1007/s00592-022-01871-6 35347427

[113] Su Y Jiang X Li Y Li F Cheng Y Peng Y Maternal low protein isocaloric diet suppresses pancreatic β-cell proliferation in mouse offspring via miR-15b. *Endocrinology.* (2016) 157:4782–93. 10.1210/en.2016-1167 27754789

[114] Cui X You L Zhu L Wang X Zhou Y Li Y Change in circulating microRNA profile of obese children indicates future risk of adult diabetes. *Metabolism.* (2018) 78:95–105. 10.1016/j.metabol.2017.09.006 28966078

[115] Zhang JT Cai QY Ji SS Zhang HX Wang YH Yan HT Decreased miR-143 and increased miR-21 placental expression levels are associated with macrosomia. *Mol Med Rep.* (2016) 13:3273–80. 10.3892/mmr.2016.4892 26934915

[116] Li B Fan J Chen NA. Novel regulator of type II diabetes: MicroRNA-143. *Trends Endocrinol Metab.* (2018) 29:380–8. 10.1016/j.tem.2018.03.019 29680463

[117] Nesca V Guay C Jacovetti C Menoud V Peyot ML Laybutt DR Identification of particular groups of microRNAs that positively or negatively impact on beta cell function in obese models of type 2 diabetes. *Diabetologia.* (2013) 56:2203–12. 10.1007/s00125-013-2993-y 23842730

[118] Liang YZ Li JJ Xiao HB He Y Zhang L Yan YX. Identification of stress-related microRNA biomarkers in type 2 diabetes mellitus: a systematic review and meta-analysis. *J Diabetes.* (2020) 12:633–44. 10.1111/1753-0407.12643 29341487

[119] Duan J Qian XL Li J Xiao XH Lu XT Lv LC miR-29a Negatively Affects Glucose-Stimulated Insulin Secretion and MIN6 Cell Proliferation via Cdc42/β-Catenin Signaling. *Int J Endocrinol.* (2019) 2019:5219782. 10.1155/2019/5219782 31662747 PMC6735210

[120] Li H Xu L Song H. MiR-29a Alleviates High Glucose-induced Inflammation and Mitochondrial Dysfunction via Modulation of IL-6/STAT3 in Diabetic Cataracts. *Curr Eye Res.* (2021) 46:1325–32. 10.1080/02713683.2021.1887272 33615922

[121] Lin HY Wang FS Yang YL Huang YH. MicroRNA-29a Suppresses CD36 to Ameliorate High Fat Diet-Induced Steatohepatitis and Liver Fibrosis in Mice. *Cells.* (2019) 8:1298. 10.3390/cells8101298 31652636 PMC6830328

[122] Zhou Y Gu P Shi W Li J Hao Q Cao X MicroRNA-29a induces insulin resistance by targeting PPARδ in skeletal muscle cells. *Int J Mol Med.* (2016) 37:931–8. 10.3892/ijmm.2016.2499 26936652 PMC4790643

[123] Zeng Y Gu P Liu K Huang P. Maternal protein restriction in rats leads to reduced PGC-1α expression via altered DNA methylation in skeletal muscle. *Mol Med Rep.* (2013) 7:306–12. 10.3892/mmr.2012.1134 23117952

[124] Xu L Huang Z Lo TH Lee JTH Yang R Yan X Hepatic PRMT1 ameliorates diet-induced hepatic steatosis via induction of PGC1α. *Theranostics.* (2022) 12:2502–18. 10.7150/thno.63824 35401831 PMC8965489

[125] Munetsuna E Yamada H Yamazaki M Ando Y Mizuno G Hattori Y Maternal fructose intake predisposes rat offspring to metabolic disorders via abnormal hepatic programming. *FASEB J.* (2021) 35:e22030. 10.1096/fj.202101276R 34748238

[126] Zheng H Dong X Liu N Xia W Zhou L Chen X Regulation and mechanism of mouse miR-130a/b in metabolism-related inflammation. *Int J Biochem Cell Biol.* (2016) 74:72–83. 10.1016/j.biocel.2016.02.021 26923288

[127] Xiao F Yu J Liu B Guo Y Li K Deng J A novel function of microRNA 130a-3p in hepatic insulin sensitivity and liver steatosis. *Diabetes.* (2014) 63:2631–42. 10.2337/db13-1689 24677715

[128] Liu J Tang T Wang GD Liu B. LncRNA-H19 promotes hepatic lipogenesis by directly regulating miR-130a/PPARγ axis in non-alcoholic fatty liver disease. *Biosci Rep.* (2019) 39:BSR20181722. 10.1042/bsr20181722 31064820 PMC6629946

[129] Wang Y Du J Niu X Fu N Wang R Zhang Y MiR-130a-3p attenuates activation and induces apoptosis of hepatic stellate cells in nonalcoholic fibrosing steatohepatitis by directly targeting TGFBR1 and TGFBR2. *Cell Death Dis.* (2017) 8:e2792. 10.1038/cddis.2017.10 28518142 PMC5520685

[130] Su S Zhao Q He C Huang D Liu J Chen F miR-142-5p and miR-130a-3p are regulated by IL-4 and IL-13 and control profibrogenic macrophage program. *Nat Commun.* (2015) 6:8523. 10.1038/ncomms9523 26436920 PMC4600756

[131] Lee H Han S Kwon CS Lee D. Biogenesis and regulation of the let-7 miRNAs and their functional implications. *Protein Cell.* (2016) 7:100–13. 10.1007/s13238-015-0212-y 26399619 PMC4742387

[132] Jiang SA. Regulator of Metabolic Reprogramming: MicroRNA Let-7. *Transl Oncol.* (2019) 12:1005–13. 10.1016/j.tranon.2019.04.013 31128429 PMC6531867

[133] Frost RJ Olson EN. Control of glucose homeostasis and insulin sensitivity by the Let-7 family of microRNAs. *Proc Natl Acad Sci U.S.A.* (2011) 108:21075–80. 10.1073/pnas.1118922109 22160727 PMC3248488

[134] Simino LAP Panzarin C Torsoni MA Ignácio-Souza LM Milanski M Torsoni AS. Maternal resistance to diet-induced obesity partially protects newborn and post-weaning male mice offspring from metabolic disturbances. *J Dev Origins Health Dis.* (2021) 12:660–70. 10.1017/s204017442000094x 33023711

[135] Herzig S Shaw RJ. AMPK: guardian of metabolism and mitochondrial homeostasis. *Nat Rev Mol Cell Biol.* (2018) 19:121–35. 10.1038/nrm.2017.95 28974774 PMC5780224

[136] Payton A Clark J Eaves L Santos HP Jr Smeester L Bangma JT Placental genomic and epigenomic signatures associated with infant birth weight highlight mechanisms involved in collagen and growth factor signaling. *Reprod Toxicol.* (2020) 96:221–30. 10.1016/j.reprotox.2020.07.007 32721520 PMC7855285

[137] Östling H Kruse R Helenius G Lodefalk M. Placental expression of microRNAs in infants born small for gestational age. *Placenta.* (2019) 81:46–53. 10.1016/j.placenta.2019.05.001 31138431

[138] Vrijens K Tsamou M Madhloum N Gyselaers W Nawrot TS. Placental hypoxia-regulating network in relation to birth weight and ponderal index: the ENVIRONAGE Birth Cohort Study. *J Transl Med.* (2018) 16:2. 10.1186/s12967-017-1375-5 29316938 PMC5761191

[139] Hussey MR Burt A Deyssenroth MA Jackson BP Hao K Peng S Placental lncRNA expression associated with placental cadmium concentrations and birth weight. *Environ Epigenetics.* (2020) 6:dvaa003. 10.1093/eep/dvaa003 32411397 PMC7211362

[140] Garcia-Beltran C Carreras-Badosa G Bassols J Malpique R Plou C de Zegher F microRNAs in newborns with low birth weight: relation to birth size and body composition. *Pediatr Res.* (2022) 92:829–37. 10.1038/s41390-021-01845-4 34799665

[141] Lin Y Zhang Y Xu L Long W Shan C Ding H High expression of an unknown long noncoding RNA RP11-290L1.3 from GDM macrosomia and its effect on preadipocyte differentiation. *Endocrine Connect.* (2021) 10:191–204. 10.1530/ec-20-0584 33475530 PMC7983522

[142] Žarković M Hufsky F Markert UR Marz M. The Role of Non-Coding RNAs in the Human Placenta. *Cells.* (2022) 11:1588. 10.3390/cells11091588 35563893 PMC9104507

[143] Thamotharan S Chu A Kempf K Janzen C Grogan T Elashoff DA Differential microRNA expression in human placentas of term intra-uterine growth restriction that regulates target genes mediating angiogenesis and amino acid transport. *PLoS One.* (2017) 12:e0176493. 10.1371/journal.pone.0176493 28463968 PMC5413012

[144] Zou Z He Z Cai J Huang L Zhu H Luo Y. Potential role of microRNA-424 in regulating ERRγ to suppress trophoblast proliferation and invasion in fetal growth restriction. *Placenta.* (2019) 83:57–62. 10.1016/j.placenta.2019.07.001 31477209

[145] Hromadnikova I Dvorakova L Kotlabova K Krofta L. The prediction of gestational hypertension, preeclampsia and fetal growth restriction via the first trimester screening of plasma exosomal C19MC microRNAs. *Int J Mol Sci.* (2019) 20:2972. 10.3390/ijms20122972 31216670 PMC6627682

[146] Higashijima A Miura K Mishima H Kinoshita A Jo O Abe S Characterization of placenta-specific microRNAs in fetal growth restriction pregnancy. *Prenatal Diagn.* (2013) 33:214–22. 10.1002/pd.4045 23354729

[147] Jing J Wang Y Quan Y Wang Z Liu Y Ding Z. Maternal obesity alters C19MC microRNAs expression profile in fetal umbilical cord blood. *Nutr Metab.* (2020) 17:52. 10.1186/s12986-020-00475-7 32655673 PMC7339545

[148] Wang D Na Q Song WW Song GY. Altered Expression of miR-518b and miR-519a in the placenta is associated with low fetal birth weight. *Am J Perinatol.* (2014) 31:729–34. 10.1055/s-0033-1361832 24683074

[149] Chen CY Chen J He L Stiles BL. PTEN. Tumor suppressor and metabolic regulator. *Front Endocrinol.* (2018) 9:338. 10.3389/fendo.2018.00338 30038596 PMC6046409

[150] Qian X Li X Shi Z Xia Y Cai Q Xu D PTEN suppresses glycolysis by dephosphorylating and inhibiting autophosphorylated PGK1. *Mol Cell.* (2019) 76:516–27.e7. 10.1016/j.molcel.2019.08.006 31492635

[151] Jiang H Wu W Zhang M Li J Peng Y Miao TT Aberrant upregulation of miR-21 in placental tissues of macrosomia. *J Perinatol.* (2014) 34:658–63. 10.1038/jp.2014.58 24786382

[152] Saha S Choudhury J Ain R. MicroRNA-141-3p and miR-200a-3p regulate insulin-like growth factor 2 during mouse placental development. *Mol Cell Endocrinol.* (2015) 414:186–93. 10.1016/j.mce.2015.07.030 26247408

[153] Tang Q Wu W Xu X Huang L Gao Q Chen H miR-141 contributes to fetal growth restriction by regulating PLAG1 expression. *PLoS One.* (2013) 8:e58737. 10.1371/journal.pone.0058737 23554918 PMC3598866

[154] Juma AR Damdimopoulou PE Grommen SV Van de Ven WJ De Groef B. Emerging role of PLAG1 as a regulator of growth and reproduction. *J Endocrinol.* (2016) 228:R45–56. 10.1530/joe-15-0449 26577933

[155] Voz ML Agten NS Van de Ven WJ Kas K. PLAG1, the main translocation target in pleomorphic adenoma of the salivary glands, is a positive regulator of IGF-II. *Cancer Res.* (2000) 60:106–13. 10646861

[156] Chen KS Stroup EK Budhipramono A Rakheja D Nichols-Vinueza D Xu L Mutations in microRNA processing genes in Wilms tumors derepress the IGF2 regulator PLAG1. *Genes Dev.* (2018) 32:996–1007. 10.1101/gad.313783.118 30026293 PMC6075147

[157] Bharathavikru R Hastie ND. Overgrowth syndromes and pediatric cancers: how many roads lead to IGF2? *Genes Dev.* (2018) 32:993–5. 10.1101/gad.317792.118 30068702 PMC6075144

[158] Chao W D’Amore PA. IGF2: epigenetic regulation and role in development and disease. *Cytokine Growth Factor Rev.* (2008) 19:111–20. 10.1016/j.cytogfr.2008.01.005 18308616 PMC2314671

[159] Dai N. The diverse functions of IMP2/IGF2BP2 in metabolism. *Trends Endocrinol Metab.* (2020) 31:670–9. 10.1016/j.tem.2020.05.007 32586768

[160] Cimmino A Calin GA Fabbri M Iorio MV Ferracin M Shimizu M miR-15 and miR-16 induce apoptosis by targeting BCL2. *Proc Natl Acad Sci U.S.A.* (2005) 102:13944–9. 10.1073/pnas.0506654102 16166262 PMC1236577

[161] Abdelmohsen K Srikantan S Kuwano Y Gorospe M. miR-519 reduces cell proliferation by lowering RNA-binding protein HuR levels. *Proc Natl Acad Sci U.S.A.* (2008) 105:20297–302. 10.1073/pnas.0809376106 19088191 PMC2629338

[162] Abdelmohsen K Srikantan S Tominaga K Kang MJ Yaniv Y Martindale JL Growth inhibition by miR-519 via multiple p21-inducing pathways. *Mol Cell Biol.* (2012) 32:2530–48. 10.1128/mcb.00510-12 22547681 PMC3434494

[163] Wang D Na Q Song G Wang Y Wang Y. The role of circRNA-SETD2/miR-519a/PTEN axis in fetal birth weight through regulating trophoblast proliferation. *Biomed Res Int.* (2020) 2020:9809632. 10.1155/2020/9809632 32626774 PMC7306081

[164] Piwecka M Glažar P Hernandez-Miranda LR Memczak S Wolf SA Rybak-Wolf A Loss of a mammalian circular RNA locus causes miRNA deregulation and affects brain function. *Science.* (2017) 357:eaam8526. 10.1126/science.aam8526 28798046

[165] Militello G Weirick T John D Döring C Dimmeler S Uchida S. Screening and validation of lncRNAs and circRNAs as miRNA sponges. *Brief Bioinform.* (2017) 18:780–8. 10.1093/bib/bbw053 27373735

[166] Zhang T Liu H Mao R Yang H Zhang Y Zhang Y The lncRNA RP11-142A22.4 promotes adipogenesis by sponging miR-587 to modulate Wnt5β expression. *Cell Death Dis.* (2020) 11:475. 10.1038/s41419-020-2550-9 32561739 PMC7305230

[167] Zhang X Li TY Xiao HM Ehrlich KC Shen H Deng HW Epigenomic and transcriptomic prioritization of candidate obesity-risk regulatory GWAS SNPs. *Int J Mol Sci.* (2022) 23:1271. 10.3390/ijms23031271 35163195 PMC8836216

[168] Tsunoda Y Kudo M Wada R Ishino K Kure S Sakatani T Expression level of long noncoding RNA H19 of normotensive placentas in late pregnancy relates to the fetal growth restriction. *J Obstetr Gynaecol Res.* (2020) 46:1025–34. 10.1111/jog.14260 32323427

[169] Zhang L Deng X Shi X Dong X. Silencing H19 regulated proliferation, invasion, and autophagy in the placenta by targeting miR-18a-5p. *J Cell Biochem.* (2019) 120:9006–15. 10.1002/jcb.28172 30536700 PMC6587755

[170] Wang Q Lu X Li C Zhang W Lv Y Wang L Down-regulated long non-coding RNA PVT1 contributes to gestational diabetes mellitus and preeclampsia via regulation of human trophoblast cells. *Biomed Pharm.* (2019) 120:109501. 10.1016/j.biopha.2019.109501 31627090

[171] Melnik BC Stremmel W Weiskirchen R John SM Schmitz G. Exosome-derived MicroRNAs of human milk and their effects on infant health and development. *Biomolecules.* (2021) 11:851. 10.3390/biom11060851 34200323 PMC8228670

[172] Nelissen EC van Montfoort AP Dumoulin JC Evers JL. Epigenetics and the placenta. *Hum Reprod Update.* (2011) 17:397–417. 10.1093/humupd/dmq052 20959349

[173] Inzaghi E Kistner A Germani D Deodati A Vanpee M Legnevall L A prospective case-control study on miRNA circulating levels in subjects born small for gestational age (SGA) evaluated from childhood into young adulthood. *PLoS One.* (2020) 15:e0228075. 10.1371/journal.pone.0228075 31978117 PMC6980597

[174] Wang H Luo C Wu X Zhang J Xu Z Liu Y Circular RNA hsa_circ_0081343 promotes trophoblast cell migration and invasion and inhibits trophoblast apoptosis by regulating miR-210-5p/DLX3 axis. *Reprod Biol Endocrinol.* (2021) 19:123. 10.1186/s12958-021-00795-0 34365964 PMC8351162

[175] Yu L Sun Y Chu Z. MiR-212-3p promotes proliferation and migration of trophoblast in fetal growth restriction by targeting placental growth factor. *Bioengineered.* (2021) 12:5655–63. 10.1080/21655979.2021.1967069 34470571 PMC8806470

